# Response surface optimization of light conditions for organic matter accumulation in two different shapes of *Arthrospira platensis*

**DOI:** 10.3389/fnut.2022.1047685

**Published:** 2023-01-06

**Authors:** Sun Jian-Fei, Shang Meng-hui, Zang Xiao-nan

**Affiliations:** Key Laboratory of Marine Genetics and Breeding, Ministry of Education, Ocean University of China, Qingdao, China

**Keywords:** *Arthrospira platensis*, shape, red and blue LED, light cycle, light intensity, response surface experiment

## Abstract

*Arthrospira platensis* has attracted wide attention as a cyanobacteria with high nutritional value. In this research, the response surface method was used to study the effects of light cycle, light intensity and red-blue LED conditions on the growth and organic matter accumulation in spiral shaped strain *A. platensis* OUC623 and linear shaped strain *A. platensis* OUC793. The light conditions suitable for *A. platensis* OUC623 were as follows: growth (light time 12.01 h, light intensity 35.64 μmol/m^2^s, LED red: blue = 6.38:1); chlorophyll a (light time 12.75 h, light intensity 31.06 μmol/m^2^s, red: blue = 6.25:1); carotenoid (light time 13.12 h, light intensity 32.25 μmol/m^2^s, red: blue = 5.79:1); polysaccharide (light time 16.00 h, light intensity 31.32 μmol/m^2^s, blue: red = 6.24:1); protein (light time 12.18 h, light intensity 6.12 μmol/m^2^s, blue: red = 7.95:1); phycocyanin (light time12.00 h, light intensity 5.00 μmol/m^2^s, blue: red = 8.00:1). The light conditions suitable for *A. platensis* OUC793 were as follows: growth (light time 13.52 h, light intensity 40.22 μmol/m^2^s, red: blue = 5.98:1); chlorophyll a (light time 14.22 h, light intensity 44.96 μmol/m^2^s, red: blue = 5.94:1); carotenoid (light time 14.13 h, light intensity 44.50 μmol/m^2^s, red: blue = 6.02:1); polysaccharide (light time 16.00 h, light intensity 31.85 μmol/m^2^s, blue: red = 6.08:1); protein (light time12.00 h, light intensity 5.00 μmol/m^2^s, blue: red = 8.00:1); phycocyanin (light time12.01 h, light intensity 5.01 μmol/m^2^s, blue: red = 8.00:1). Under the theoretical optimal light conditions, compared with white LED, the growth rate, chlorophyll a, carotenoid, phycocyanin, protein and polysaccharide contents in strain 623 increased by 91.67%, 114.70%, 85.05%, 563.54%, 386.14%, 201.18%, and in strain 793 increased by 75.00%, 150.94%, 113.43%, 427.09%, 1284.71%, 312.38%, respectively. The two strains showed different advantages. Growth rate, chlorophyll a, polysaccharide, protein and phycocyanin content of strain 623 were higher than those of strain 793, while carotenoid was higher in strain 793. After optimization, both strains could reach a good growth state, and the growth rate and organic matter content were close. And then a 20 L photobioreactor was used to expand the culture of the two strains, validating the theoretical optimal light conditions of response surface method. This study laid the foundation for the establishment of optical conditions for organic matter accumulation in two different strains of *A. platensis*, which provided more options for meeting the industrialization needs of *A. platensis*.

## 1. Introduction

*Arthrospira platensis* belongs to Cyanophyta, Cyanophyceae, Oscillatoriales, Oscillatoriales and *Arthrospira*. Due to the high nutritional value, *A. platensis* has been widely concerned and studied. *A. platensis* stands out for being one of the richest protein sources of microbial origin, with a protein content of 60–70% of cell dry weight. Proteins extracted from *A. platensis* have high nutritional quality when compared to proteins of animal and vegetable origin (1). Many kinds of amino acids, especially lysine and threonine were found in *A. platensis*, which are necessary for humans and animals (2). The phycobilisomes of *A. platensis* are composed of allophycocyanin (APC) and phycocyanin (CPC) which account for 60% of the total protein content (3). Among the proteins of *A. platensis*, phycocyanin is the most abundant, which is not only a protein with complete amino acid composition, but also a natural pigment. Phycocyanin has also been proven to have anti-tumor effects (4). Chlorophyll a and carotenoid are also the main pigments in *A. platensis.* The content of chlorophyll a in *A. platensis* is three times higher than that of most terrestrial plants, which is very similar to human hemoglobin and can be used as the direct raw material of human hemoglobin (5). Chlorophyll a and iron ions have significant effects on tonifying blood, hematopoiesis, strengthening and activating tissue cells (5). Numerous studies have shown that a diet rich in carotenoids has positive health effects, helping to reduce the risk of age-related diseases such as cancer, cardiovascular disease, skin or eye diseases, and mental health during pregnancy and metabolic health is also of great benefit in early years, some carotenoids, in addition to acting as colorants and bioactive compounds beneficial to human health, have well-defined vitamin A nutritional roles and are essential in the fight against vitamin A deficiency (6, 7). Carotenoid has antioxidant properties, inhibits the synthesis of low-density lipoprotein, prevents the destruction of blood cells, and reduces the occurrence of thrombosis and myocardial infarction. However, human body cannot synthesize carotenoid and only get it from food (8). *A. platensis* also contains other high-value compounds such as polysaccharide. Polysaccharides are the main form of carbohydrates in *A. platensis*, which can promote blood circulation, activate the production of hormones in the body, promote epinephrine and insulin secretion, and improve the reaction speed of the nervous system (9). Carbohydrates account for 15–20%, mainly represented by glucan (10). In general, the nutrients in A. platensis are rich and have important physiological functions, and also have good anti-tumor, anti-viral and anti-radiation effects, so they are widely used in food and medicine (11).

Light is one of the most important environmental factors affecting the growth, cell morphology and accumulation of intracellular metabolites of microalgae. In recent years, there are many studies on the effect of light conditions for *A. platensis* culture. Xue et al. (12) found that in a certain range, the growth rate of algae was proportional to the light intensity, but when it reached light saturation, the yield of *A. platensis* almost did not change with the increase of light intensity. Markou (13) studied the effects of various color of LED light on the growth and organic matter accumulation of *A. platensis*. It was found that red light was the most effective light in algae photosynthesis, and the combination of a small amount of other LED light was more conducive to the accumulation of organic matter. Tian et al. (14) studied the effects of red and blue LED light on the production of phycocyanin by photosynthetically active radiation of *Spirulina/A. platensis*. It was found that red and blue LED had different effects on the growth and organic matter accumulation of *A. platensis* under fixed light intensity. Blue light is beneficial to the synthesis of phycocyanin, while red light is beneficial to photosynthesis and promote the accumulation of growth rate. Blue light: red light = 3:1 is most suitable for the production of phycocyanin, and the combination of red and blue LED is significantly better than traditional fluorescent light on the growth and organic matter accumulation in algae (14). However, there is still a lack of systematic research on the combination of light conditions for the culture of different shapes of *A. platensis* and the accumulation of phycocyanin, polysaccharide and other organic compounds.

The traditional culture methods of *A. platensis* were open-air culture, the problems of which were as follows: the culture process was seriously restricted by environmental and weather factors, the light was uneven and insufficient, the quality of *A. platensis* varies greatly in different culture batches, and the algae liquid cultured in the open-air was easy to be polluted. So that the quality and yield of *A. platensis* were difficult to control. In recent years, photobioreactor has been used to cultivate *A. platensis*, which can well control the cultivation conditions, ensure the uniform and stable illumination and prevent the pollution of the algae, so that the quality and yield of *A. platensis* have been improved. In the previous reports, it is found that different light conditions have different effects on the biomass, pigment and organic matter accumulation of *A. platensis* (12–14). Therefore, optimizing the suitable light conditions for culture and organic matter accumulation in *A. platensis* will help to set up different light conditions to meet the industrial needs.

In this experiment, two strains of *A. platensis* OUC623 and *A. platensis* OUC793 were selected to study. Because of their fast growth rate and good adaptability. The morphology of strain *A. platensis* OUC623 is spiral and strain *A. platensis* OUC793 is linear, which has obvious morphological differences, so it is easy to understand the light requirements of different shapes of *A. platensis.* In this experiment, the response surface experiment of light cycle, light intensity and red-blue LED light combination was carried out, and the optimum light condition was obtained by taking the growth rate, pigment, polysaccharide, phycocyanin and protein content of algae as indexes, which provided experimental basis for the industrial application of *A. platensis*.

## 2. Materials and methods

### 2.1. Algae species and culture conditions

#### 2.1.1. Algae species

*Arthrospira platensis* OUC623 and *A. platensis* OUC793 were bred by the Algae Genetics Laboratory of Ocean University of China.

**FIGURE 1:**
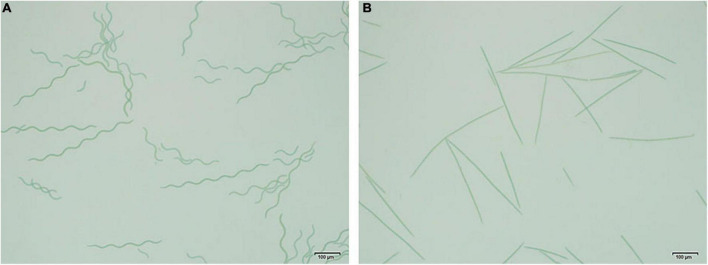
Morphological photograph of *Arthrospira platensis.* **(A)** Morphological photograph of *A. platensis* OUC623. **(B)** Morphological photograph of *A. platensis* OUC793.

The shape of OUC623 was a regular spiral curve, with the length of the filament of 416.89 ± 18.98 μm, the pitch of 77.31 ± 2.07 μm, and the spiral width of 22.31 ± 1.17 μm (Figure 1A). OUC793 was a linear rod, without spiral curve, the length of the filament was 425.47 ± 18.50 μm (Figure 1B).

#### 2.1.2. Culture medium and culture conditions

In this experiment, *A. platensis* was cultured in Zarrouk medium (15). The culture temperature was 25°C. 150 mL liquid medium and 10 mL activated algae solution were added to the 250 mL conical flask, and the initial culture concentration of the algae solution was OD_560_ = 0.2. The algae were shaken three times a day to ensure the uniformity of the system.

### 2.2. Experimental method

#### 2.2.1. Lighting condition setting

Before the response surface experiment, single factor experiments were carried out under three illumination conditions, respectively, including light cycle, combination of red and blue LED light and light intensity. The light cycle was set as L8:D16, L10:D14, L12:D12, L14:D10, L16:D8, the LED light combination were white light, red: blue = 1:1, red: blue = 2:1, red: blue = 4:1, red: blue = 6:1, red: blue = 8:1, and blue: red = 1:1, blue: red = 2:1, blue: red = 4:1, blue: red = 6:1, blue: red = 8:1, and the light intensity gradients was set as 5, 15, 25, 35, and 45 μmol/m^2^s. The initial light intensity of white light is 25 μmol/m^2^s. Culture temperature was 25°C.

The spectral range of white light, red light and blue light is 390–760, 622–760, and 435–450 nm, respectively.

#### 2.2.2. Determination of growth rate

According to the reference (16), standard curve was made between OD_560_ and *A. platensis* cell mass. At the end of logarithmic growth (day12), 3 mL of the shaken algal liquid was taken to measure the OD_560_, and the growth rate was obtained according to the regression equation (1).


(1) Dry⁢weight⁢of⁢algae⁢(g/L⋅day)

$$=\left(0.6427\times {\mathrm{OD}}_{560}+0.1463\right)/12\left(R^{2}=0.9916>0.735\right)$$

#### 2.2.3. Determination of pigment concentration

According to reference (17) and slightly improved, 5 ml of the shaken *A. platensis* liquid was centrifuged and removed supernatant, then broken for 15 min after adding 5 mL 80% acetone solution. The algae liquid was incubated at room temperature for at least 12 h then clarified by centrifugation for 5 min at 15,000 g. The absorption values of OD_663_, OD_646_, and OD_470_ were measured in spectrophotometer, and the contents of chlorophyll a and carotenoid (mg/g) per unit mass of algae were calculated according to the formulas (2) and (3) and the concentration (g/L) of algal solution. Ca in the formula (3) means the concentration of chlorophyll a (mg/L).


(2) Concentration⁢of⁢chlorophylla⁢(mg/L)

$$=12.21\times {\mathrm{OD}}_{663}-2.81\times {\mathrm{OD}}_{646}$$

(3) Concentration⁢of⁢carotenoid⁢(mg/L)

$$=\left(1000\times {\mathrm{OD}}_{470}-3.27\times \mathrm{Ca}\right)/229$$

#### 1.2.4. Determination of polysaccharide concentration

According to the reference (18), the content of polysaccharide was determined by sulfuric acid-anthrone method, and the standard curve was made with glucose. 5 ml of the shaken *A. platensis* liquid was centrifuged and removed supernatant. After cell fragmentation for 10 min in 5 ml 0.01 M PBS buffer, 20 ml 0.1% anthrone sulfate reagent was added and the absorbance at OD_620_ was measured by spectrophotometer. According to the standard curve (4) and concentration (g/L) of the algal solution. The content of polysaccharide (mg/g) per unit mass of algae was calculated.


(4) Polysaccharide⁢content⁢(mg/ml)

$$=1.3934\times OD_{620}-0.0104\left(R^{2}=0.9985>0.735\right)$$

#### 2.2.5. Determination of protein content

According to the improved Lowry (19) method, the standard curve was made with bovine serum albumin. 5 ml of the shaken *A. platens*is liquid was centrifuged and removed supernatant. After *A. platensis* was broken for 10 min in 5 ml 0.01 M PBS buffer, 20 ml Coomassie brilliant blue reagent was added, mixed gently, and left to stand for 5 min, and the absorbance at OD_595_ was measured in spectrophotometer. According to the standard curve (5) and the concentration of algal solution (g/L). The protein content (mg/g) per unit mass of algae was calculated.


(5) Protein⁢content⁢(μ⁢g/ml)

$$=161.52\times OD_{595}-12.391\left(R^{2}=0.9941>0.735\right)$$

#### 2.2.6. Determination of phycocyanin content

According to the reference (20) about PC (phycocyanin) content determination method, 5 ml of the shaken *A. platens*is liquid was centrifuged and removed supernatant. After *A. platensis* was broken for 10 min in 5 ml 0.01 M PBS buffer, the absorbance values of OD_620_ and OD_652_ were measured in spectrophotometer, and the phycocyanin content was calculated by formula (6). Then the phycocyanin content (mg/g) per unit mass of algae was calculated according to the concentration of algal solution (g/L).


$$\mathrm{PC}\,\left(\mathrm{mg}/\mathrm{ml}\right)=\left({\mathrm{OD}}_{620}-0.474\times {\mathrm{OD}}_{652}\right)/5.43$$

#### 2.2.7. Response surface experiment design

Based on the results of single factor experiments, the growth and organic matter accumulation of two kinds of algae under different light cycle, light intensity and LED light combination were analyzed, and three levels were selected, respectively. Taking the growth rate, pigment content, protein content, phycocyanin and polysaccharide content as response values, the response surface test of three factors and three levels was designed by using software Design Expert 12 (21).

#### 2.2.8. Verification of response surface experiment and expand culture of algae

The results of response surface optimization were verified in the flask. All the samples were set up according to the optimum conditions optimized by response surface methodology.

And also, a 20 L photobioreactor was used to verify the optimized light conditions of response surface and expand culture of algae strains. The photobioreactor is composed of a double-layer hollow acrylic tank with a height of 1.5 m and a volume of 20 L. It is equipped with controllable LED illumination system, using central illumination mode, which can adjust the ratio of red and blue LED light, light intensity and light time according to the needs of *A. platensis* growth and organic matter accumulation. The airlift air supply system is adopted, the aeration plate is located at the bottom of the tank, and the ventilation rate is 3 L/min. The temperature control system keeps the temperature in the reactor at 25°C.

#### 2.2.9. Statistics and analysis of data

All experiments were set up with three parallel samples, each sample was tested for three replicates. Statistical analysis was conducted using IBM SPSS Statistics (Version 22) to determine the effect of individual factor on the growth and organic matter accumulation in strains *A. platensis* OUC623 and *A. platensis* OUC793 of light cycle, light intensity and red-blue LED light conditions (single-factor experiment). If the *P*-value was less than 0.05, the difference was statistically significant, on the contrary, the difference was not significant. Design-Expert software (Version 12) was used to construct Box–Behnken design and assess the effect of linear and quadratic interactions of the operational parameters on the accumulation of the growth and organic matter (22).

## 3. Result analysis

### 3.1. Effect of light cycle on *A. platensis*

The light cycle was set as L8:D16, L10:D14, L12:D12, L14:D10, L16:D8, and *A. platensis* OUC623 and *A. platensis* OUC793 were cultured under white LED light with light intensity of 25 μmol/m^2^s. Culture temperature was 25°C.

As seen from Table 1, the growth rate of *A. platensis* OUC623 and *A. platensis* OUC793 increased at first and then decreased with the extension of the light time in the light cycle. For strain 623, when the illumination time was 12–16 h, the growth rate was higher, which were 0.12 ± 0.01 g/L⋅day (L12:D12), 0.10 ± 0.01 g/L⋅day (L14:D10), 0.10 ± 0.01 g/L⋅day (L16:D8), respectively. While the light cycle had no significant effect on the growth rate of strain 793, the growth rates were similar at about 0.09–0.12 g/L⋅day under 8 to 16 h of illumination in a day.

**TABLE 1:** Growth rate and organic matter content of *Arthrospira platensis* OUC623 and *A. platensis* OUC793 under different light cycle.

Strain	Light cycle (h)	Growth rate	Chlorophyll a content	Carotenoid content	Polysaccharide content	Protein content	Phycocyanin content
623	L8:D16	0.09 ± 0.01 g/L⋅day^a^	2.11 ± 0.24 mg/g ^a^	2.28 ± 0.22 mg/g^a^	64.52 ± 1.26 mg/g^a^	87.89 ± 1.11 mg/g^a^	8.44 ± 0.61 mg/g^a^
	L10:D14	0.09 ± 0.01 g/L⋅day^a^	3.16 ± 0.24 mg/g ^ab^	2.54 ± 0.11 mg/g^a^	92.36 ± 1.25 mg/g^bc^	120.34 ± 0.16 mg/g^b^	8.94 ± 1.02 mg/g^a^
	L12:D12	0.12 ± 0.01 g/L⋅day^b^	4.15 ± 1.10 mg/g ^b^	3.21 ± 0.34 mg/g^b^	99.99 ± 4.11 mg/g^c^	144.47 ± 2.97 mg/g^c^	11.52 ± 0.44 mg/g^b^
	L14:D10	0.10 ± 0.01 g/L⋅day^ab^	4.11 ± 0.92 mg/g ^b^	3.15 ± 0.21 mg/g^b^	90.24 ± 3.24 mg/g^b^	130.15 ± 6.54 mg/g^d^	10.25 ± 0.33 mg/g^b^
	L16:D8	0.10 ± 0.01 g/L⋅day^ab^	3.54 ± 0.24 mg/g ^b^	3.01 ± 0.11 mg/g^b^	78.21 ± 8.21 mg/g^d^	110.25 ± 3.14 mg/g^e^	8.51 ± 0.11 mg/g^a^
793	L8:D16	0.09 ± 0.01 g/L⋅day^a^	2.11 ± 0.11 mg/g ^a^	1.89 ± 0.28 mg/g^a^	40.40 ± 3.21 mg/g^a^	41.25 ± 2.45 mg/g^a^	11.24 ± 0.45 mg/g^a^
	L10:D14	0.09 ± 0.01 g/L⋅day^a^	2.54 ± 0.15 mg/g ^ab^	2.19 ± 0.16 mg/g^a^	51.24 ± 1.24 mg/g^b^	42.15 ± 4.56 mg/g^a^	12.21 ± 0.22 mg/g^b^
	L12:D12	0.12 ± 0.02 g/L⋅day^a^	3.20 ± 0.87 mg/g ^b^	2.83 ± 0.21 mg/g^b^	66.48 ± 2.41 mg/g^c^	50.55 ± 2.21 mg/g^b^	14.10 ± 0.31 mg/g^c^
	L14:D10	0.10 ± 0.03 g/L⋅day^a^	3.21 ± 0.14 mg/g ^b^	2.22 ± 0.43mg/g^a^	64.13 ± 1.58 mg/g^c^	50.65 ± 0.14 mg/g^b^	11.24 ± 0.12 mg/g^a^
	L16:D8	0.09 ± 0.03 g/L⋅day^a^	3.00 ± 0.21 mg/g ^b^	2.01 ± 0.10mg/g^a^	58.45 ± 2.17 mg/g^d^	49.17 ± 1.03 mg/g^b^	9.45 ± 0.11 mg/g^d^

The same letters in the same column indicate that there is no significant difference between groups (*P* > 0.05), but different letters indicate significant differences between groups (*P* < 0.05).

In terms of pigment accumulation, the chlorophyll a content of *A. platensis* OUC623 and *A. platensis* OUC793 was higher under the condition of light time longer than 10 h in the light cycle, and it was 4.15 ± 1.10 mg/g (L12:D12, strain 623), 4.11 ± 0.92 mg/g (L14:D10, strain 623), 3.54 ± 0.24 mg/g (L16:D8, strain 623), and 3.20 ± 0.87 mg/g (L12:D12, strain 793), 3.21 ± 0.14 mg/g (L14:D10, strain 793), 3.00 ± 0.21 mg/g (L16:D8, strain 793) after 12 days of culture.

The carotenoid accumulation of strain 623 was higher under the condition of light time longer than 10 h in the light cycle, and the contents were 3.21 ± 0.34 mg/g (L12:D12), 3.15 ± 0.21 mg/g (L14:D10), 3.01 ± 0.11 mg/g (L16:D8) on the 12th day of culture. The carotenoid accumulation of strain 793 was the highest under the photoperiod L12:D12, and the contents was 2.83 ± 0.21 mg/g on the 12th day of culture.

The polysaccharide content of strains 623 and 793 increased at first and then decreased with the extension of light time in photoperiod. The polysaccharide content of strains 623 was the highest under the condition of L10:D14 (92.36 ± 1.25 mg/g) and L12:D12 (99.99 ± 4.11 mg/g). The polysaccharide content of strain 793 was the highest under the condition of L12:D12 (66.48 ± 2.41 mg/g) and L14:D10 (64.13 ± 1.58 mg/g).

The protein and phycocyanin content also increased at first and then decreased with the extension of light time in the photoperiod. The protein content of strain 623 was the highest under the photoperiod L12:D12, which was 144.47 ± 2.97 mg/g. And the protein content of the strain 793 was higher under the photoperiod longer than 10 h in the light cycle, which were 50.55 ± 2.21 mg/g (L12:D12), 50.65 ± 0.14 mg/g (L14:D10), 49.17 ± 1.03 mg/g (L16:D8). The phycocyanin content of strain 623 was the highest under the condition of photoperiod L12:D12 and L14:D10, which was 11.52 ± 0.44 mg/g and 10.25 ± 0.33 mg/g, respectively. The phycocyanin content of strain 793 was the highest under the condition of photoperiod L12:D12, which was 14.10 ± 0.31 mg/g.

### 3.2. Effects of different red-blue LED on *A. platensis*

The light combination of LED was white light, red: blue = 1:1, red: blue = 2:1, red: blue = 4:1, red: blue = 6:1, red: blue = 8:1, with the light intensity of 25 μmol/m^2^s and light cycle of L12:D12. Culture temperature was 25°C.

As shown in Table 2, the growth rate of strains 623 and 793 increased at first and then showed a decreasing trend with the increase of red-light ratio. The growth rate of strain 623 was higher when red: blue = 4:1–8:1 at about 0.19–0.21 g/L⋅day. The growth rate of strain 793 was higher when red: blue = 2:1–8:1 at about 0.17–0.19 g/L⋅day.

**TABLE 2:** Growth rate and organic matter content of *Arthrospira platensis* OUC623 and *A. platensis* OUC793 under different red-blue LED light combination.

Strain	Red-Blue LED light combination	Growth rate	Chlorophyll a content	Carotenoid content	Polysaccharide content	Protein content	Phycocyanin content
623	White LED	0.10 ± 0.01 g/L⋅day^a^	4.95 ± 0.67 mg/g^a^	4.71 ± 0.29 mg/g^a^	100.24 ± 1.36 mg/g^a^	142.24 ± 3.33 mg/g^a^	12.63 ± 0.07 mg/g^a^
	01:01	0.16 ± 0.02 g/L⋅day^b^	7.44 ± 0.53 mg/g^cd^	5.62 ± 0.41 mg/g^bc^	207.11 ± 4.42 mg/g^d^	280.12 ± 8.21 mg/g^b^	39.42 ± 5.24 mg/g^b^
	02:01	0.17 ± 0.02g/L⋅day^bc^	7.10 ± 0.53 mg/g^bc^	5.34 ± 0.42 mg/g^abc^	172.19 ± 5.64 mg/g^c^	266.39 ± 1.87 mg/g^c^	32.34 ± 3.07 mg/g^c^
	04:01	0.19 ± 0.02 g/L⋅day^bcd^	7.63 ± 0.64 mg/g^cd^	4.96 ± 0.47 mg/g^ab^	164.33 ± 2.88 mg/g^c^	225.40 ± 2.31 mg/g^d^	23.31 ± 1.43 mg/g^d^
	06:01	0.21 ± 0.0 2g/L⋅day^d^	8.53 ± 0.67 mg/g^d^	5.86 ± 0.47 mg/g^c^	115.75 ± 4.23 mg/g^b^	129.59 ± 4.21 mg/g^e^	11.15 ± 0.45 mg/g^a^
	08:01	0.20 ± 0.02g/L⋅day^cd^	6.11 ± 0.55 mg/g^ab^	5.41 ± 0.45 mg/g^bc^	100.02 ± 5.64 mg/g^a^	121.43 ± 2.11 mg/g^e^	14.24 ± 2.86 mg/g^a^
793	White LED	0.12 ± 0.01g/L⋅day^a^	4.57 ± 0.51 mg/g^a^	5.48 ± 0.35 mg/g^ab^	64.58 ± 0.31 mg/g^a^	57.86 ± 3.44 mg/g^a^	13.58 ± 0.62 mg/g^a^
	01:01	0.15 ± 0.01 g/L⋅day^ab^	5.68 ± 0.48 mg/g^b^	4.95 ± 0.29 mg/g^a^	90.15 ± 3.29 mg/g^b^	161.15 ± 3.45 mg/g^b^	34.99 ± 5.16 mg/g^b^
	02:01	0.17 ± 0.02g/L⋅day^bc^	6.49 ± 0.52 mg/g^b^	5.69 ± 0.35 mg/g^b^	85.28 ± 5.93 mg/g^b^	105.77 ± 4.51 mg/g^c^	25.61 ± 1.15 mg/g^c^
	04:01	0.17 ± 0.02 g/L⋅day^bc^	7.81 ± 0.55 mg/g^c^	6.53 ± 0.32 mg/g^c^	85.42 ± 6.12 mg/g^b^	91.84 ± 1.08 mg/g^d^	21.12 ± 0.91 mg/g^c^
	06:01	0.19 ± 0.02 g/L⋅day^c^	8.27 ± 0.52 mg/g^cd^	5.61 ± 0.29 mg/g^b^	70.12 ± 2.21 mg/g^a^	59.92 ± 1.54 mg/g^a^	10.84 ± 0.17 mg/g^a^
	08:01	0.18 ± 0.02 g/L⋅day^bc^	9.24 ± 0.66 mg/g^d^	6.56 ± 0.32 mg/g^c^	65.48 ± 3.21 mg/g^a^	46.89 ± 2.03 mg/g^e^	12.24 ± 1.99 mg/g^a^

The same letters in the same column indicate that there is no significant difference between groups (*P* > 0.05), but different letters indicate significant differences between groups (*P* < 0.05).

The content of chlorophyll a of strain 623 under LED red: blue = 1: 1–6: 1 were higher. When the culture reached the plateau stage, the content of chlorophyll a of strain 623 was about 7.10–8.53 mg/g. The content of chlorophyll a of strain 793 was the highest when red: blue = 6:1, 8:1, which was 8.27 ± 0.52 mg/g, 9.24 ± 0.66 mg/g, respectively.

The content of carotenoid of strain 623 under LED red: blue = 1: 1–8: 1 were similar. When the culture reached the plateau stage, the content of carotenoid of strain 623 were 4.96–5.86 mg/g. The content of carotenoid of strain 793 was the highest when red: blue = 4:1, 8:1, which was 6.53 ± 0.32 mg/g, 6.56 ± 0.32 mg/g, respectively.

The results of polysaccharide content showed that proper proportion of red and blue LED light combinations could increase the polysaccharide accumulation in *A. platensis*. The highest polysaccharide content of strain 623 (207.11 ± 4.42 mg/g) was obtained when the LED light combinations of red: blue = 1:1. The higher polysaccharide content of strain 793 was 90.15 ± 3.29 mg/g, 85.28 ± 5.93 mg/g, 85.42 ± 6.12 mg/g under red: blue = 1:1, 2:1, 4:1, respectively. With the further increase of the proportion of red light, the polysaccharide content of the two algae strains showed a downward trend.

Protein and phycocyanin content of *A. platensis* also increased under the proper proportion of red and blue LED light (red: blue = 1:1, 2:1, 4:1), which were significantly higher than that under white light (*P* < 0.05). When the LED light combinations at red: blue = 1:1, the protein content of strains 623 and 793 was the highest, which was 280.12 ± 8.21 mg/g and 161.15 ± 3.45 mg/g, respectively; and the phycocyanin content of strain 623 and 793 was 39.42 ± 5.24 mg/g and 34.99 ± 5.16 mg/g, respectively. With the further increase of the proportion of red light, the protein content of the two algae strains showed a downward trend.

### 3.3. Effects of different proportion of blue-red LED on *A. platensis*

LED light combination was set as: white light, blue: red = 1:1, blue: red = 2:1, blue: red = 4:1, blue: red = 6:1, blue: red = 8:1 with light intensity of 25 μmol/m^2^s, light cycle of L12:D12. Culture temperature was 25°C.

As shown in Table 3, a certain proportion of blue-red LED could promote the growth of strain 623. However, with the increase of the proportion of blue light, the growth rate of strain 623 showed a downward trend. The growth rate of the strain 623 was the highest when blue: red = 1: 1, 2: 1 which were 0.17 ± 0.01 g/L⋅day and 0.16 ± 0.01 g/L⋅day, respectively. The growth rate of the strain 793 was also promoted at blue: red = 1: 1 slightly and showed a downward trend with the increase of the proportion of blue light, however, the difference was not significant compared with white light.

**TABLE 3:** Growth rate and organic matter content of *Arthrospira platensis* OUC623 and *A. platensis* OUC793 under different blue-red LED light combination.

Strain	Blue-Red LED light combination	Growth rate	Chlorophyll a content	Carotenoid content	Polysaccharide content	Protein content	Phycocyanin content
623	White LED	0.10 ± 0.01 g/L⋅day^a^	3.67 ± 0.81 mg/g^a^	1.75 ± 0.17 mg/g^a^	111.54 ± 12.35 mg/g^a^	152.23 ± 10.09 mg/g^a^	10.91 ± 0.21 mg/g^a^
	01:01	0.17 ± 0.01 g/L⋅day^b^	7.53 ± 0.44 mg/g^b^	5.01 ± 0.11 mg/g^b^	202.71 ± 1.25 mg/g^b^	284.79 ± 4.92 mg/g^b^	31.38 ± 1.96 mg/g^b^
	02:01	0.16 ± 0.01 g/L⋅day^b^	6.63 ± 0.44 mg/g^bc^	4.09 ± 0.34 mg/g^c^	210.75 ± 13.21 mg/g^bc^	351.37 ± 27.83 mg/g^c^	32.16 ± 0.61 mg/g^b^
	04:01	0.14 ± 0.01 g/L⋅day^c^	6.42 ± 0.39 mg/g^c^	3.85 ± 0.24 mg/g^c^	227.87 ± 5.44 mg/g^cd^	516.75 ± 3.11 mg/g^d^	49.29 ± 2.31 mg/g^c^
	06:01	0.14 ± 0.01 g/L⋅day^c^	5.74 ± 0.36 mg/g^c^	3.98 ± 0.28 mg/g^c^	240.49 ± 6.54 mg/g^d^	630.44 ± 12.41 mg/g^e^	51.41 ± 1.24 mg/g^c^
	08:01	0.12 ± 0.01 g/L⋅day^d^	4.27 ± 0.24 mg/g^a^	1.85 ± 0.12 mg/g^a^	200.53 ± 7.21 mg/g^b^	661.56 ± 24.25 mg/g^e^	53.55 ± 4.21 mg/g^c^
793	White LED	0.12 ± 0.01 g/L⋅day^ab^	5.16 ± 0.67 mg/g^a^	2.43 ± 0.26 mg/g^a^	49.57 ± 2.77 mg/g^a^	58.87 ± 16.02 mg/g^a^	24.34 ± 1.17 mg/g^a^
	01:01	0.14 ± 0.01 g/L⋅day^b^	7.52 ± 0.92 mg/g^b^	4.31 ± 0.55 mg/g^d^	90.71 ± 2.18 mg/g^b^	159.16 ± 9.21 mg/g^b^	38.71 ± 3.11 mg/g^b^
	02:01	0.13 ± 0.01 g/L⋅day^ab^	8.64 ± 0.79 mg/g^bc^	3.52 ± 0.34 mg/g^c^	99.39 ± 2.04 mg/g^bc^	433.12 ± 23.54 mg/g^c^	61.19 ± 1.51 mg/g^c^
	04:01	0.12 ± 0.01 g/L⋅day^ab^	9.13 ± 0.71 mg/g^c^	3.45 ± 0.3 2mg/g^c^	107.86 ± 8.22 mg/g^c^	499.46 ± 3.27 mg/g^d^	66.66 ± 1.21 mg/g^d^
	06:01	0.12 ± 0.01 g/L⋅day^ab^	8.39 ± 0.67mg/g ^bc^	3.15 ± 0.26 mg/g^bc^	149.56 ± 12.05 mg/g^d^	624.65 ± 7.57 mg/g^e^	75.46 ± 3.21 mg/g^e^
	08:01	0.11 ± 0.01 g/L⋅day^a^	8.41 ± 0.67 mg/ ^bc^	2.61 ± 0.26 mg/g^ab^	188.54 ± 3.66 mg/g^e^	627.75 ± 38.29 mg/g^e^	69.56 ± 4.29 mg/g^d^

The same letters in the same column indicate that there is no significant difference between groups (*P* > 0.05), but different letters indicate significant differences between groups (*P* < 0.05).

The chlorophyll a content of strain 623 decreased with the increase of the proportion of blue light, and the highest value (7.53 ± 0.44 mg/g, 6.63 ± 0.44 mg/g) was at the LED light combination blue: red = 1: 1 and 2: 1, respectively. The chlorophyll a content of strain 793 showed a trend of increase at first and then decrease with the increase of the proportion of blue light, but the differences were not significant at blue: red = 2: 1–8: 1, with the content of 8.39–9.13 mg/g. The carotenoid content of strains 623 and 793 decreased with the increase of the proportion of blue light. The carotenoid accumulation of the two algae strains was the highest when the LED light combination was blue: red = 1:1, which was 5.01 ± 0.11 mg/g and 4.31 ± 0.55 mg/g, respectively.

The polysaccharide content of strains 623 and 793 increased with the increase of the proportion of blue light. The polysaccharide content of strains 623 and 793 under 1:1–8:1 blue and red LED was significantly higher than that of white LED light with the same light conditions (*P* < 0.05). When strain 623 was in blue: red = 4:1, 6:1 and strain 793 was in blue: red = 8:1, the polysaccharide content was the highest (227.87 ± 5.44 mg/g, 240.49 ± 6.54 mg/g, and 188.54 ± 3.66 mg/g, respectively).

The protein and phycocyanin content of strains 623 and 793 both increased with the increase of the proportion of blue light, and the content under blue: red = 1:1–8:1 was higher than that under white light with significant difference (*P* < 0.05). When LED blue: red = 6: 1, 8: 1, the protein content of strains 623 and 793 was the highest, which was 630.44 ± 12.41 mg/g, 661.56 ± 24.25 mg/g, and 624.65 ± 7.57 mg/g, 627.75 ± 38.29 mg/g, respectively. When LED light combination was blue: red = 4: 1–8: 1, the phycocyanin content of strain 623 was higher, which was 49.29 ± 2.31 mg/g (blue: red = 4: 1), 51.41 ± 1.24 mg/g (blue: red = 6: 1), 53.55 ± 4.21 mg/g (blue: red = 8: 1), and when LED light combination was blue: red = 6:1, the phycocyanin content of strain 793 was the highest (75.46 ± 3.21 mg/g).

### 2.4. Effects of different light intensity on *A. platensis*

According to the results of 2.2 and 2.3, the LED light combination of red: blue = 6:1, which can significantly promote growth and the LED light combination of blue: red = 6:1, which can significantly promote the accumulation of organic matter were selected to study the light intensity. The light intensity gradients were set as 5, 15, 25, 35, 45 μmol/m^2^s with light cycle of L12:D12. Culture temperature was 25°C.

As shown in Table 4, under red: blue = 6:1 and blue: red = 6:1, the growth rates of strains 623 and 793 both showed upward trend with the increase of light intensity, and reached the highest when the light intensity was 25–45 μmol/m^2^s. The higher growth rate of strain 623 under the LED light combination of red: blue = 6:1 was 0.20 ± 0.01 g/L⋅day (25 μmol/m^2^s), 0.21 ± 0.01 g/L⋅day (35 μmol/m^2^s), 0.23 ± 0.02 g/L⋅day (45 μmol/m^2^s), respectively; and under blue: red = 6:1, when light intensity was 45 μmol/m^2^s, the highest growth rate was 0.19 ± 0.01 g/L⋅day. For strain 793, regardless red: blue = 6:1 or blue: red = 6:1, the higher growth rates all appeared under the light intensity of 25–45 μmol/m^2^s, which was 0.19 ± 0.02 g/L⋅day (red: blue = 6:1, 25 μmol/m^2^s), 0.20 ± 0.02 g/L⋅day (red: blue = 6:1, 35 μmol/m^2^s), 0.22 ± 0.02 g/L⋅day (red: blue = 6:1, 45 μmol/m^2^s), and 0.11 ± 0.02 g/L⋅day (blue: red = 6:1, 25 μmol/m^2^s), 0.15 ± 0.03 g/L⋅day (blue: red = 6:1, 35 μmol/m^2^s), 0.15 ± 0.02 g/L⋅day (blue: red = 6:1, 45 μmol/m^2^s).

**TABLE 4:** Growth rate and organic matter content of *Arthrospira platensis* OUC623 and *A. platensis* OUC793 under different light intensity.

Strain	LED light combination	light intensity	Growth rate	Chlorophyll a content	Carotenoid content	Polysaccharide content	Protein content	Phycocyanin content
623	Red:Blue = 6:1	5 mmol/m^2^s	0.07 ± 0.01 g/L⋅day^a^	3.48 ± 0.13 mg/g^a^	2.35 ± 0.20 mg/g^a^	40.21 ± 2.11 mg/g^a^	190.21 ± 4.15 mg/g^a^	12.54 ± 0.21mg/g ^a^
		15 mmol/m^2^⋅	0.11 ± 0.03 g/L⋅day^b^	5.11 ± 0.21 mg/g^b^	3.14 ± 0.32 mg/g^b^	84.57 ± 1.25 mg/g^b^	174.21 ± 5.48 mg/g^b^	10.51 ± 0.54mg/g ^b^
		25 mmol/m^2^s	0.20 ± 0.01 g/L⋅day^c^	5.55 ± 0.31 mg/g^b^	2.91 ± 0.32 mg/g^ab^	111.54 ± 6.45 mg/g^c^	111.89 ± 3.11 mg/g^c^	10.55 ± 0.15 mg/g^b^
		35 mmol/m^2^s	0.21 ± 0.01 g/L⋅day^c^	6.15 ± 0.12 mg/g^c^	3.22 ± 0.36 mg/g^b^	88.45 ± 4.05 mg/g^b^	106.32 ± 2.44 mg/g^c^	8.44 ± 0.14 mg/g^c^
		45 mmol/m^2^⋅s	0.23 ± 0.02 g/L⋅day^c^	6.54 ± 0.31 mg/g^c^	3.05 ± 0.36mg/g ^b^	85.45 ± 3.69mg/g ^b^	90.14 ± 6.45mg/g ^d^	7.01 ± 0.22mg/g ^d^
623	Blue:Red = 6:1	5 mmol/m^2^s	0.04 ± 0.01 g/L⋅day^a^	2.99 ± 0.11 mg/g^a^	0.90 ± 0.06 mg/g^a^	210.44 ± 5.45 mg/g^a^	666.15 ± 5.14 mg/g^a^	58.15 ± 1.21 mg/g^a^
		15 mmol/m^2^s	0.05 ± 0.01 g/L⋅day ^a^	3.89 ± 0.21 mg/g^b^	1.05 ± 0.11 mg/g^a^	211.56 ± 6.78 mg/g^a^	621.11 ± 1.71 mg/g^b^	50.12 ± 2.14 mg/g^b^
		25 mmol/m^2^s	0.14 ± 0.01 g/L⋅day^b^	6.55 ± 0.32 mg/g^c^	3.05 ± 0.28 mg/g^b^	220.45 ± 6.45 mg/g^a^	580.12 ± 6.44 mg/g^c^	50.45 ± 1.88 mg/g^b^
		35 mmol/m^2^s	0.16 ± 0.02 g/L⋅day^b^	5.14 ± 0.51 mg/g^d^	3.05 ± 0.28 mg/g^b^	300.15 ± 7.58 mg/g^b^	481.10 ± 3.14 mg/g^d^	48.11 ± 2.01 mg/g^b^
		45 mmol/m^2^s	0.19 ± 0.01 g/L⋅day^c^	6.45 ± 0.45 mg/g^c^	3.21 ± 0.34 mg/g^b^	322.14 ± 8.45 mg/g^c^	394.12 ± 5.54 mg/g^e^	44.19 ± 2.22 mg/g^c^
793	Red:Blue = 6:1	5 mmol/m^2^s	0.06 ± 0.01 g/L⋅day ^a^	4.51 ± 0.64 mg/g ^a^	1.33 ± 0.15mg/g ^a^	42.15 ± 4.11mg/g ^a^	89.45 ± 4.54mg/g ^a^	16.45 ± 0.24mg/g ^a^
		15 mmol/m^2^s	0.15 ± 0.02 g/L⋅day ^b^	6.11 ± 0.34 mg/g^b^	2.67 ± 0.26 mg/g^b^	51.48 ± 3.14 mg/g^b^	65.48 ± 1.64 mg/g^b^	11.24 ± 0.12 mg/g^b^
		25 mmol/m^2^s	0.19 ± 0.02 g/L⋅day ^c^	8.00 ± 0.48 mg/g^cd^	3.48 ± 0.36 mg/g^c^	66.45 ± 2.15 mg/g^c^	55.44 ± 1.45 mg/g^c^	9.88 ± 0.31 mg/g^c^
		35 mmol/m^2^s	0.20 ± 0.02 g/L⋅day^c^	8.97 ± 0.64 mg/g^d^	3.81 ± 0.38 mg/g^c^	74.78 ± 3.15 mg/g^d^	38.15 ± 2.54 mg/g^d^	8.48 ± 0.12 mg/g^d^
		45 mmol/m^2^s	0.22 ± 0.02 g/L⋅day^c^	7.45 ± 0.47 mg/g^c^	3.06 ± 0.32 mg/g^c^	49.15 ± 1.11 mg/g^b^	22.45 ± 0.12 mg/g^e^	4.15 ± 0.14 mg/g^e^
793	Blue:Red = 6:1	5 mmol/m^2^s	0.04 ± 0.02 g/L⋅day^a^	3.11 ± 0.22 mg/g^a^	0.59 ± 0.05 mg/g^a^	59.14 ± 4.18 mg/g^a^	694.15 ± 9.23 mg/g^a^	60.22 ± 0.24 mg/g^a^
		15 mmol/m^2^s	0.06 ± 0.01 g/L⋅day ^a^	4.15 ± 0.42 mg/g ^a^	1.10 ± 0.12mg/g ^a^	77.15 ± 2.44 mg/g ^b^	689.45 ± 2.26mg/g ^a^	69.15 ± 0.64mg/g ^b^
		25 mmol/m^2^s	0.11 ± 0.02 g/L⋅day^b^	7.89 ± 0.80 mg/g^c^	3.13 ± 0.35mg/g^b^	153.14 ± 3.15 mg/g^c^	601.45 ± 1.28 mg/g^b^	68.12 ± 1.88 mg/g^b^
		35mmol/m^2^s	0.15 ± 0.03 g/L⋅day^b^	7.01 ± 0.79 mg/g^bc^	3.15 ± 0.35 mg/g^b^	201.45 ± 5.12mg/g ^d^	587.45 ± 1.17 mg/g^c^	21.45 ± 2.14 mg/g^c^
		45 mmol/m^2^s	0.15 ± 0.02 g/L⋅da ^b^	6.51 ± 0.14 mg/g^b^	3.11 ± 0.34 mg/g^b^	222.65 ± 3.45 mg/g^e^	546.12 ± 0.88 mg/g^d^	29.44 ± 3.16 mg/g^d^

The same letters in the same column indicate that there is no significant difference between groups (*P* > 0.05), but different letters indicate significant differences between groups (*P* < 0.05).

The changes of pigment content of strains 623 and 793 under different light conditions were similar. No matter red: blue = 6:1 or blue: red = 6:1, the content of chlorophyll a and carotenoid was lower under the light intensity of 5–25 μmol/m^2^s, and then showed an upward trend with the increase of light intensity. Under red: blue = 6:1, the chlorophyll a content of strain 623 reached the highest at 35 μmol/m^2^s (6.15 ± 0.12 mg/g) and 45 μmol/m^2^s (6.54 ± 0.31 mg/g), and the carotenoid content was higher at 15–45 μmol/m^2^s, about 2.91–3.22 mg/g. Under blue: red = 6:1, the chlorophyll a content and carotenoid content of strain 623 both reached the highest at 25–45 μmol/m^2^s, about 5.14–6.55 and 3.05–3.21 mg/g, respectively. For strain 793, under red: blue = 6:1, the chlorophyll a content reached the highest at 25 μmol/m^2^s (8.00 ± 0.48 mg/g) and 35 μmol/m^2^s (8.97 ± 0.64 mg/g), and the carotenoid content was higher at 25–45 μmol/m^2^s, about 3.06–3.81 mg/g. Under blue: red = 6:1, the chlorophyll a content reached the highest at 25 μmol/m^2^s (7.89 ± 0.80 mg/g) and 35 μmol/m^2^s (7.01 ± 0.79 mg/g), and the carotenoid content was higher at 25–45μmol/m^2^s, about 3.11–3.15 mg/g.

Under the condition of red: blue = 6:1, the polysaccharide content of algae strains 623 and 793 increased at first and then decreased with the increase of light intensity. The polysaccharide content of strain 623 reached the highest (111.54 ± 6.4 5mg/g) when the light intensity was 25 μmol/m^2^s. The polysaccharide content of strain 793 reached the highest (74.78 ± 3.15 mg/g) when the light intensity was 35 μmol/m^2^s. Under the LED light condition of blue: red = 6:1, the polysaccharide content of strains 623 and 793 increased gradually with the increase of light intensity, and the polysaccharide content of strain 623 was the highest when the light intensity was 45 μmol/m^2^s, which was 322.14 ± 8.45 mg/g. The polysaccharide content of strain 793 was the highest at 45 μmol/m^2^s, which was 222.65 ± 3.45 mg/g.

The results of protein content showed that no matter red: blue = 6:1 or blue: red = 6:1, the protein content of strains 623 and 793 decreased with the increase of light intensity, and the highest was at the light intensity of 5–15 μmol/m^2^s. Under the light intensity of 5 μmol/m^2^s, the protein content of strain 623 was 190.21 ± 4.15 mg/g (under red: blue = 6:1) and 666.15 ± 5.14 mg/g (under blue: red = 6:1); and the protein content of strain 793 was 89.45 ± 4.54 mg/g (under red: blue = 6:1), and 694.15 ± 9.23 mg/g (under blue: red = 6:1), 689.45 ± 2.26 mg/g (under blue: red = 6:1, light intensity of 15 μmol/m^2^s).

The results of phycocyanin content of strain 623 showed that when the LED light combination of red: blue = 6:1 and blue: red = 6:1, the phycocyanin content was the highest at the light intensity of 5 μmol/m^2^s, and the phycocyanin content was 12.54 ± 0.21 mg/g (red: blue = 6:1) and 58.15 ± 1.21 mg/g (blue: red = 6:1), respectively. The results of phycocyanin content of strain 793 showed that when the LED light combination of red: blue = 6:1, the phycocyanin content was the highest (16.45 ± 0.24 mg/g) at the light intensity of 5 μmol/m^2^s. When the LED light combination of blue: red = 6:1, the content of phycocyanin was the highest at 15 and 25 μmol/m^2^s, which was 69.15 ± 0.64 and 68.12 ± 1.88 mg/g.

### 3.5. Optimization of light cycle, light intensity and LED light combination conditions by response surface method

Based on the comprehensive analysis of the growth and organic matter accumulation of two strains of *A. platensis* in the single factor experiments in 2.1–2.4, three levels of light cycle (L12:D12, L14:D10, L16:D8), light intensity (5, 15, 25, 35, 45 μmol/m^2^s) and LED light combination (red4: blue1, red6: blue1, red8: blue1; blue4: red1, blue6: red1, blue8: red1) were selected. The growth rate, pigment content, protein content, phycocyanin content and polysaccharide content of *A. platensis* OUC623 and *A. platensis* OUC793 were used as the response values, and the response surface test of 3 factors and 3 levels was designed with the software Design Expert 12. The experimental factors and levels are shown in Tables 5–7. The culture temperature was 25°C.

**TABLE 5:** Response surface experimental design and results of growth rate and pigment accumulation of *Arthrospira platensis* OUC623 and *A. platensis* OUC793.

Strain	Group	Light time in the light cycle	Light intensity	LED light combination	Growth rate	chlorophyll a content	carotenoid content
		**(h)**	**(μmol/m^2^s)**	**Red:Blue**	**(g/L⋅day)**	**(mg/g)**	**(mg/g)**
623	1	14	35	6	0.196	8.095	3.187
	2	14	35	6	0.199	7.941	3.126
	3	16	45	6	0.185	4.784	2.357
	4	12	35	4	0.162	5.530	2.406
	5	16	35	4	0.139	3.456	1.812
	6	14	25	4	0.088	5.260	2.032
	7	14	35	6	0.210	8.184	3.222
	8	14	45	4	0.090	3.391	1.904
	9	12	35	8	0.163	5.845	1.950
	10	16	25	6	0.102	3.125	1.891
	11	14	35	6	0.199	8.103	3.190
	12	14	35	6	0.199	8.014	3.155
	13	12	25	6	0.152	8.067	2.834
	14	12	45	6	0.196	5.474	2.151
	15	14	25	8	0.147	4.859	1.948
	16	16	35	8	0.152	3.179	1.913
	17	14	45	8	0.137	3.720	0.847
793	1	14	35	6	0.184	4.976	2.890
	2	14	35	6	0.181	4.881	2.835
	3	16	45	6	0.165	5.157	3.044
	4	12	35	4	0.152	2.375	1.334
	5	16	35	4	0.112	2.464	1.408
	6	14	25	4	0.118	4.211	2.579
	7	14	35	6	0.186	5.030	2.922
	8	14	45	4	0.143	4.632	2.926
	9	12	35	8	0.142	2.141	1.104
	10	16	25	6	0.097	3.158	2.164
	11	14	35	6	0.184	4.981	2.893
	12	14	35	6	0.182	4.926	2.861
	13	12	25	6	0.161	3.041	2.009
	14	12	45	6	0.164	5.390	3.152
	15	14	25	8	0.099	3.829	1.923
	16	16	35	8	0.138	2.273	1.276
	17	14	45	8	0.170	4.018	2.261

**TABLE 6:** Response surface experimental design and results of polysaccharide accumulation of *Arthrospira platensis* OUC623 and *A. platensis* OUC793.

Strain	Group	Light time in the light cycle	Light intensity	LED light combination	polysaccharide content
		**(h)**	**(μmol/m^2^s)**	**Blue: Red**	**(mg/g)**
623	1	14	35	6	116.846
	2	14	35	6	114.624
	3	16	45	6	115.597
	4	12	35	4	89.954
	5	16	35	4	83.807
	6	14	25	4	79.366
	7	14	35	6	118.133
	8	14	45	4	83.579
	9	12	35	8	85.129
	10	16	25	6	128.821
	11	14	35	6	116.963
	12	14	35	6	115.676
	13	12	25	6	78.190
	14	12	45	6	102.230
	15	14	25	8	78.226
	16	16	35	8	99.728
	17	14	45	8	81.064
793	1	14	35	6	109.058
	2	14	35	6	106.984
	3	16	45	6	120.014
	4	12	35	4	81.112
	5	16	35	4	93.612
	6	14	25	4	78.741
	7	14	35	6	110.259
	8	14	45	4	80.987
	9	12	35	8	78.063
	10	16	25	6	124.551
	11	14	35	6	109.168
	12	14	35	6	107.967
	13	12	25	6	69.539
	14	12	45	6	100.667
	15	14	25	8	72.392
	16	16	35	8	97.173
	17	14	45	8	80.320

**TABLE 7:** Response surface experimental design and results of protein and phycocyanin accumulation of *Arthrospira platensis* OUC623 and *A. platensis* OUC793.

Strain	Group	Light time in the light cycle	Light intensity	LED light combination	protein content	phycocyanin content
		**(h)**	**(μmol/m^2^s)**	**Blue: Red**	**(mg/g)**	**(mg/g)**
623	1	14	15	6	607.005	59.880
	2	14	15	6	609.966	58.882
	3	14	15	6	604.044	57.884
	4	14	15	6	602.070	59.381
	5	14	15	6	601.083	59.481
	6	14	25	8	663.264	65.868
	7	14	5	8	701.757	76.846
	8	14	25	4	491.526	30.938
	9	14	5	4	563.577	51.896
	10	16	15	8	671.160	67.864
	11	12	15	8	677.082	70.858
	12	16	15	4	505.344	39.920
	13	12	15	4	534.954	44.910
	14	16	25	6	552.720	50.000
	15	12	25	6	572.460	55.888
	16	16	5	6	611.940	61.876
	17	12	5	6	657.342	62.874
793	1	14	15	6	631.680	48.483
	2	14	15	6	621.810	45.177
	3	14	15	6	627.732	46.279
	4	14	15	6	630.693	51.789
	5	14	15	6	633.654	45.574
	6	14	25	8	660.303	57.298
	7	14	5	8	711.627	74.929
	8	14	25	4	504.357	34.159
	9	14	5	4	601.083	45.177
	10	16	15	8	691.887	65.012
	11	12	15	8	702.744	69.419
	12	16	15	4	556.668	36.362
	13	12	15	4	580.356	39.668
	14	16	25	6	591.213	49.585
	15	12	25	6	609.966	42.974
	16	16	5	6	632.667	50.687
	17	12	5	6	645.498	55.095

#### 3.5.1. Growth rate

According to the analysis of the growth rate of single factor experiment, *A. platensis* OUC623 and *A. platensis* OUC793 were cultured under red and blue LED light at three factors and three levels, including light cycle (L12:D12; L14:D10; L16:D8), light intensity (25; 35; 45 μmol/m^2^s), LED light combination (red 4: blue 1; red 6: blue 1; red 8: blue 1). The experimental design and results are shown in Table 5.

**FIGURE 2:**
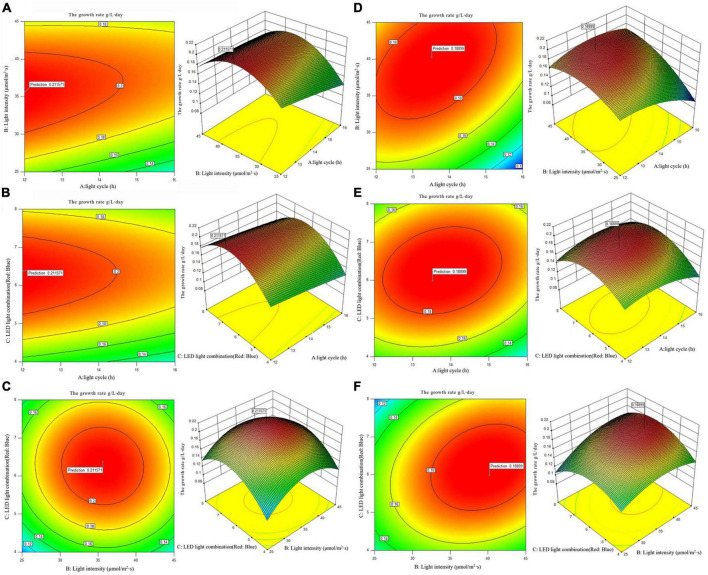
2D contour diagram and 3D surface diagram of growth rate of *Arthrospira platensis* OUC623 and *A. platensis* OUC793. **(A)** Contour diagram and response surface diagram of growth rate of 623 with light time (12, 14, and 16 h) and light intensity (25, 35, and 45 μmol/m^2^s) under the light of red6: blue 1. **(B)** Contour diagram and response surface diagram of growth rate of 623 with light time (12, 14, and 16 h) and red and blue light combination (red: blue = 4:1, 6:1, 8:1) under light intensity of 35 μmol/m^2^s. **(C)** Contour diagram and response surface diagram of growth rate of 623 with red and blue light combination (red: blue = 4:1, 6:1, 8:1) and light intensity (25, 35, and 45 μmol/m^2^s) under the light cycle of L14:D10. **(D)** Contour diagram and response surface diagram of growth rate of 793 with light time (12, 14, and 16 h) and light intensity (25, 35, and 45 μmol/m^2^s) under the light of red6: blue 1. **(E)** Contour diagram and response surface diagram of growth rate of 793 with light time (12, 14, and 16 h) and red and blue light combination (red: blue = 4:1, 6:1, 8:1) under light intensity of 35 μmol/m^2^s. **(F)** Contour diagram and response surface diagram of growth rate of 793 with red and blue light combination (red: blue = 4:1, 6:1, 8:1) and light intensity (25, 35, and 45 μmol/m^2^s) under the light cycle of L14:D10.

In order to reflect the effects of various experimental factors and their interactions on the growth rate of the two algae strains, the 2D contour map and 3D response surface maps were drawn by software analysis, and the results are shown in Figure 2.

When the fixed LED light combination is red: blue = 6: 1, the growth rate of strain 623 decreases with the increase of light time, increases at first and then decreases with the increase of light intensity, the growth rate of strain 793 increases at first and then decreases with the increase of light time and light intensity. The contour map of strain 623 is oval, but the *p*-value = 0.4149 > 0.05, indicating that the interaction between light cycle and light intensity of strain 623 is not significant (Figure 2A). The contour map of strain 793 is oval, and the *p*-value = 0.0002 < 0.01, indicating that the interaction between light cycle and light intensity of strain 793 is highly significant (Figure 2D).

When the fixed light intensity is 35 μmol/m^2^s, it is found that the growth rate of strain 623 decreases with the increase of light time, increases at first and then decreases with the increase of the proportion of red light, the growth rate of strain 793 increases at first and then decreases with the increase of light time and the proportion of red light. The contour map of strain 623 is oval, but the *p*-value = 0.7974 > 0.05, indicating that there is a non-significant interaction between light cycle and LED light combination of strain 623 (Figure 2B). The contour map of strain 793 is oval, and the *p*-value = 0.0056 < 0.01, indicating that there is a very significant interaction between light cycle and LED light combination of strain 793 (Figure 2E).

When the light cycle is L14:D10, it can be found that the growth rate of strains 623 and 793 increases at first and then decreases with the increase of the proportion of red light and light intensity. The contour map of strain 623 is roughly circular, and the *p*-value = 0.7974 > 0.05, indicating that the interaction between light intensity and LED light combination is not significant (Figure 2C). The contour map of strain 793 is oval, and the *p*-value = 0.0015 < 0.01, indicating that the interaction between light intensity and LED light combination is very significant (Figure 2F).

The order of primary and secondary effects of light cycle, light intensity and light combination on growth rate is as follows: strain 623, light combination (*F*-value = 3.55) > light intensity (*F*-value = 3.5) > light cycle (*F*-value = 2.23); strain 793, light combination (*F*-value = 166.63) > light cycle (*F*-value = 76.91) > light intensity (*F*-value = 68.40).

After response surface optimization, the theoretical optimal culture scheme for the growth of *A. platensis* OUC623 and *A. platensis* OUC793 is shown in Table 8. When the light intensity is 35.64 μmol/m^2^s and the light time is 12.01 h, LED light combination is red: blue = 6.38:1, the theoretical maximum value of growth rate of strain 623 is 0.21 g/L⋅day. When the light intensity is 40.22 μmol/m^2^s and the light time is 13.52 h, LED light combination is red: blue = 5.98:1, the theoretical maximum value of growth rate of strain 793 is 0.19 g/L⋅day. The theoretical values of growth rate of strains 623 and 793 are 76.67 and 57.50% higher than those under white LED light, respectively.

**TABLE 8:** Theoretical optimal culture scheme for growth rate and organic matter accumulation of *Arthrospira platensis* OUC623 and *A. platensis* OUC793.

Strain	Item	Light time in the Light cycle (h)	Light intensity (mmol/m^2^s)	LED light combination	Theoretical value
623	Growth rate	12.01	35.64	Red: Blue = 6.38:1	0.21 g/L⋅day
	Chlorophyll a content	12.75	31.06	Red: Blue = 6.25:1	8.58 mg/g
	Carotenoid content	13.12	32.25	Red: Blue = 5.79:1	3.25 mg/g
	Polysaccharide content	16	31.32	Blue: Red = 6.24:1	126.60 mg/g
	Protein content	12.18	6.12	Blue: Red = 7.95:1	709.18 mg/g
	Phycocyanin content	12	5	Blue: Red = 8.00:1	75.58 mg/g
793	Growth rate	13.52	40.22	Red: Blue = 5.98:1	0.19 g/L ⋅day
	Chlorophyll a content	14.22	44.96	Red: Blue = 5.94:1	6.10 mg/g
	Carotenoid content	14.13	44.5	Red: Blue = 6.02:1	3.58 mg/g
	Polysaccharide content	16	31.85	Blue: Red = 6.08:1	124.73 mg/g
	Protein content	12	5	Blue: Red = 8.00:1	711.40 mg/g
	Phycocyanin content	12.01	5.01	Blue: Red = 8.00:1	74.94 mg/g

#### 3.5.2. The content of pigment

**FIGURE 3:**
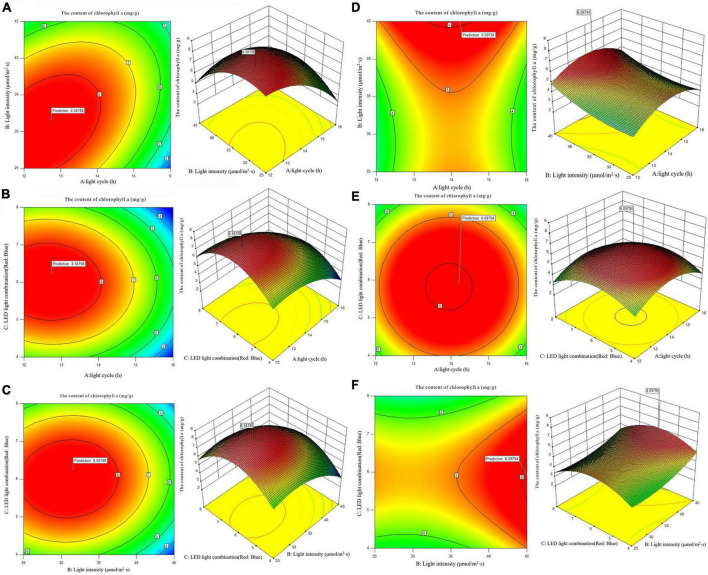
2D contour diagram and 3D surface diagram of chlorophyll a content of *Arthrospira platensis* OUC623 and *A. platensis* OUC793. **(A)** Contour diagram and response surface diagram of chlorophyll a content of 623 with light time (12, 14, and 16 h) and light intensity (25, 35, and 45 μmol/m^2^s) under the light of red6:blue 1. **(B)** Contour diagram and response surface diagram of chlorophyll a content of 623 with light time (12, 14, and 16 h) and red and blue light combination (red: blue = 4:1, 6:1, 8:1) under light intensity of 35 μmol/m^2^s. **(C)** Contour diagram and response surface diagram of chlorophyll a content of 623 with red and blue light combination (red: blue = 4:1, 6:1, 8:1) and light intensity (25, 35, and 45 μmol/m^2^s) under the light cycle of L14:D10. **(D)** Contour diagram and response surface diagram of chlorophyll a content of 793 with light time (12, 14, and 16 h) and light intensity (25, 35, and 45 μmol/m^2^s) under the light of red6:blue 1. **(E)** Contour diagram and response surface diagram of chlorophyll a content of 793 with light time (12, 14, and 16 h) and red and blue light combination (red: blue = 4:1, 6:1, 8:1) under light intensity of 35 μmol/m^2^s. **(F)** Contour diagram and response surface diagram of chlorophyll a content of 793 with red and blue light combination (red: blue = 4:1, 6:1, 8:1)and light intensity (25, 35, and 45 μmol/m^2^s)under the light cycle of L14:D10.

**FIGURE 4:**
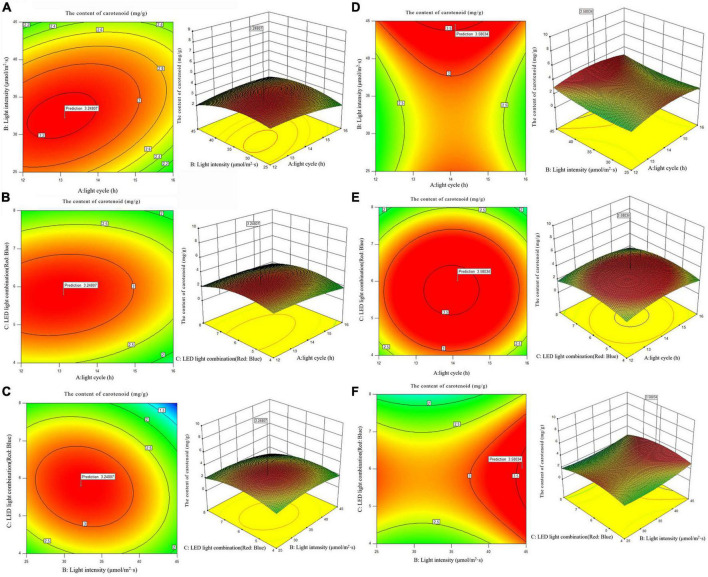
2D contour diagram and 3D surface diagram of carotenoid content of *Arthrospira platensis* OUC623 and *A. platensis* OUC793. **(A)** Contour diagram and response surface diagram of carotenoid content of 623 with light time (12, 14, and 16 h) and light intensity (25, 35, and 45 μmol/m^2^s) under the light of red6:blue 1. **(B)** Contour diagram and response surface diagram of carotenoid content of 623 with light time (12, 14, and 16 h) and red and blue light combination (red: blue = 4:1, 6:1, 8:1) under light intensity of 35 μmol/m^2^s. **(C)** Contour diagram and response surface diagram of carotenoid content of 623 with red and blue light combination (red: blue = 4:1, 6:1, 8:1) and light intensity (25, 35, and 45 μmol/m^2^s) under the light cycle of L14:D10. **(D)** Contour diagram and response surface diagram of carotenoid content of 793 with light time (12, 14, and 16 h) and light intensity (25, 35, and 45 μmol/m^2^s) under the light of red6:blue 1. **(E)** Contour diagram and response surface diagram of carotenoid content of 793 with light time (12, 14, and 16 h) and red and blue light combination (red: blue = 4:1, 6:1, 8:1) under light intensity of 35 μmol/m^2^s. **(F)** Contour diagram and response surface diagram of carotenoid content of 793 with red and blue light combination (red: blue = 4:1, 6:1, 8:1) and light intensity (25, 35, and 45 μmol/m^2^s) under the light cycle of L14:D10.

According to the analysis of the Chlorophyll a and carotenoid content of single factor experiment, three factors and three levels of light cycle (L12: D12, L14: D10, L16:D8), light intensity (25; 35; 45 μmol/m^2^s), and LED light combination (red 4: blue 1; red 6: blue 1; red 8: blue 1) were selected to culture *A. platensis* OUC623 and *A. platensis* OUC793, respectively. The content of chlorophyll a and carotenoid was used as the response value. The experimental design and results are shown in Table 5. 2D contour map and 3D response surface map about the effect of experimental factors and their interaction on chlorophyll a and carotenoid accumulation of the two strains are shown in Figures 3, 4.

##### 3.5.2.1. The content of chlorophyll a

When the fixed LED light combination is red: blue = 6:1, the chlorophyll a accumulation in strain 623 and 793 both increases at first and then decreases with the increase of light time (Figures 3A, D). With the increase of light intensity from 25 to 45 μmol/m^2^s, the chlorophyll a accumulation of strain 623 increase at first and then decrease (Figure 3A), while strain 793 keeps a rising trend (Figure 3D). The contour map of strain 623 is oval, and the *p*-value = 0.0002 < 0.01, indicating that there is a very significant interaction between light time and light intensity in strain 623. The contour map of strain 793 is oval, but the *p*-value = 0.7411 > 0.05, indicating that there is a non-significant interaction between light time and light intensity in strain 793.

When the light intensity is 35 μmol/m^2^s, it is found that the chlorophyll a accumulation of strains 623 and 793 increases at first and then decreased with the increase of light time and light intensity. The interaction is not significant between light cycle and LED light combination in strain 623 (*p*-value = 0.3716) and strain 793 (*p*-value = 0.9675) (Figures 3B, E).

When the light cycle is L14:D10, it can be found that the chlorophyll a accumulation of strain 623 increases at first and then decreases with the increase of the proportion of red light in the LED light combination and light intensity (Figure 3C). In strain 793, the chlorophyll a accumulation increases at first and then decreases with the increase of the proportion of red light, and increases with the increase of light intensity (Figure 3F). There is no significant interaction between light intensity and LED light combination in strain 623 (*p*-value = 0.2776) and strain 793 (*p*-value = 0.8263).

The order of primary and secondary effects of light cycle, light intensity and light combination on the accumulation of chlorophyll a is as follows: strain 623, light cycle (*F*-value = 139.85) > light intensity (*F*-value = 20.20) > light combination (*F*-value = 0.0015); strain 793, light intensity (*F*-value = 11.85) > light combination (*F*-value = 0.97) > light cycle (*F*-value = 0.0053).

After response surface optimization, the theoretical optimal culture scheme for the accumulation of chlorophyll a in *A. platensis* OUC623 and *A. platensis* OUC793 is shown in Table 8. When the light intensity is 31.06 μmol/m^2^s and the light time is 12.75 h, LED light combination is red: blue = 6.25:1, the theoretical maximum value of chlorophyll a of strain 623 is 8.58 mg/g. When the light intensity is 44.96 μmol/m^2^s and the light time is 14.22 h, LED light combination is red: blue = 5.94:1, the theoretical maximum value of chlorophyll a of strain 793 is 6.10 mg/g. The theoretical values of chlorophyll a of strains 623 and 793 are 106.80 and 90.56% higher than those under white LED light, respectively.

##### 3.5.2.2. The content of carotenoid

When the fixed LED light combination is red: blue = 6:1, the carotenoid accumulation of strain 623 increases at first and then decreases with the increase of light time and light intensity. The contour map of strain 623 is oval, and the *p*-value = 0.0131 < 0.05, indicating that there is a significant interaction between light cycle and light intensity (Figure 4A). The carotenoid accumulation in strain 793 increases with the increase of light intensity, first increases and then decreases with the increase of light time (Figure 4D). The contour map of strain 793 is oval, but the *p*-value = 0.5739 > 0.05, indicating that there is a non-significant interaction between light cycle and light intensity.

When the light intensity is 35 μmol/m^2^s, it is found that the carotenoid accumulation of strains 623 and 793 increases at first and then decreases with the increase of light time and the proportion of red light. The contour maps of strain 623 and strain 793 are almost oval, but the *p*-value (strain 623) = 0.1536 > 0.05 and *p*-value (strain 793) = 0.8324 > 0.05, indicating that there is a non-significant interaction between light cycle and LED light combination (Figures 4B, E).

When the light cycle is L14:D10, it can be found that the carotenoid accumulation of strain 623 increases at first and then decreases with the increase of the proportion of red light and light intensity. The contour map of strain 623 is oval, and the *p*-value = 0.0267 < 0.05, indicating that there is a significant interaction between light intensity and LED light combination (Figure 4C). In strain 793, the carotenoid accumulation increases at first and then decreases with the increase of the proportion of red light, and increases with the increase of light intensity (Figure 4F). The contour map of strain 793 is oval, but the *p*-value = 0.9845 > 0.05, indicating that there is a non-significant interaction between light intensity and LED light combination.

The order of primary and secondary effects of light cycle, light intensity and light combination on the accumulation of carotenoids is as follows: strain 623, light combination (*F*-value = 9.24) > light intensity (*F*-value = 8.63) > light cycle (*F*-value = 7.72), strain 793 light intensity (*F*-value = 18.43) > light combination (*F*-value = 7.12) > light cycle (*F*-value = 0.22).

After response surface optimization, the theoretical optimal culture scheme for carotenoid accumulation in *A. platensis* OUC623 and *A. platensis* OUC793 is shown in Table 8. When the light intensity is 32.25 μmol/m^2^s and the light time is 13.12 h, LED light combination is red: blue = 5.79:1, the theoretical maximum value of carotenoid of strain 623 is 3.25 mg/g. When the light intensity is 44.50 μmol/m^2^s and the light time is 14.13 h, LED light combination is red: blue = 6.02:1, the theoretical maximum value of carotenoid of strain 793 is 3.58 mg/g. The theoretical values of carotenoid of strains 623 and 793 are 1.2 and 26.50% higher than those under white LED light, respectively.

#### 3.5.3. The content of polysaccharide

**FIGURE 5:**
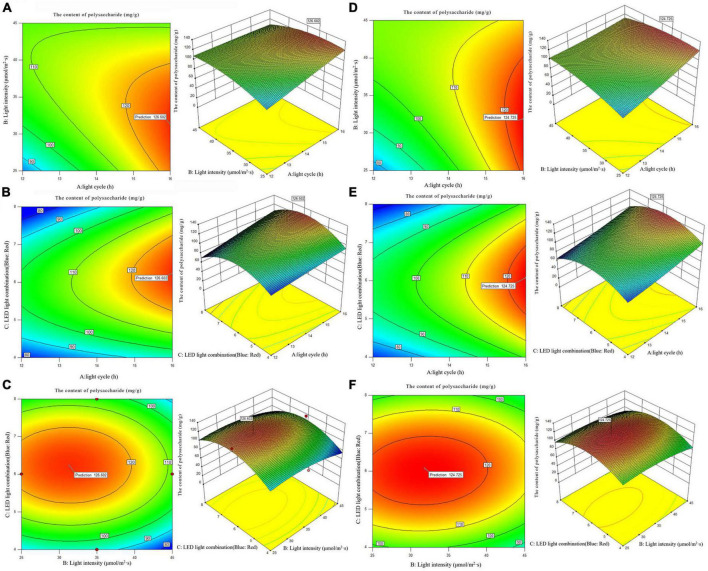
2D contour diagram and 3D surface diagram of polysaccharide content of *Arthrospira platensis* OUC623 and *A. platensis* OUC793. **(A)** Contour diagram and response surface diagram of polysaccharide content of 623 with light time (12, 14, and 16 h) and light intensity (25,35,45μmol/m^2^s) under the light of blue6:red1. **(B)** Contour diagram and response surface diagram of polysaccharide content of 623 with light time (12, 14, and 16 h) and red and blue light combination (blue: red = 4:1, 6:1, 8:1) under light intensity of 35 μmol/m^2^s. **(C)** Contour diagram and response surface diagram of polysaccharide content of 623 with red and blue light combination (blue: red = 4:1, 6:1, 8:1) and light intensity (25, 35, and 45 μmol/m^2^s) under the light cycle of L14:D10. **(D)** Contour diagram and response surface diagram of polysaccharide content of 793 with light time (12, 14, and 16 h) and light intensity (25, 35, and 45 μmol/m^2^s) under the light of blue6:red1. **(E)** Contour diagram and response surface diagram of polysaccharide content of 793 with light time (12, 14, and 16 h) and red and blue light combination (blue: red = 4:1, 6:1, 8:1) under light intensity of 35 μmol/m^2^s. **(F)** Contour diagram and response surface diagram of polysaccharide content of 793 with red and blue light combination (blue: red = 4:1, 6:1, 8:1) and light intensity (25, 35, and 45 μmol/m^2^s) under the light cycle of L14:D10.

According to the analysis of the results of single factor experiment, three factors and three levels of light cycle (L12: D12, L14: D10, L16:D8), light intensity (25; 35; 45 μmol/m^2^s), and LED light combination (blue 4: red 1; blue 6: red 1; blue 8: red 1) were selected to culture *A. platensis* OUC623 and *A. platensis* OUC793, respectively. The content of polysaccharide was used as the response value. The experimental design and results are shown in Table 6. 2D contour map and 3D response surface map were drawn to reflect the effect of experimental factors and their interaction on polysaccharide accumulation of two strains of algae, and the results are shown in Figure 5.

When the fixed LED light combination is blue: red = 6: 1, the polysaccharide accumulation of strains 623 and 793 increased with the extension of light time, and increases at first and then decreases with the increase of light intensity (Figures 5A, D). The contour maps of strain 623 (*p*-value = 0.0475 < 0.05) and strain 793 (*p*-value = 0.0249 < 0.05) both are oval, indicating that there is a significant interaction between light cycle and light intensity.

When the light intensity is 35 μmol/m^2^s, the accumulation of polysaccharide in strains 623 and 793 increases with the increase of light time, and increases at first and then decreases with the increase of the proportion of blue light in the LED light combination (Figures 5B, E). The interaction between light cycle and LED light combination is not significant in strain 623 (*p*-value = 0.2233) and strain 793 (*p*-value = 0.6146).

When the light cycle is L14:D10, the accumulation of polysaccharide in strains 623 and 793 under blue LED light first increases and then decreases with the increase of the proportion of blue light and light intensity (Figures 5C, F). There is a non-significant interaction between light intensity and LED light combination in strain 623 (*p*-value = 0.9319) and strain 793 (*p*-value = 0.6643).

The order of primary and secondary effects of light cycle, light intensity and light combination on the accumulation of polysaccharides is as follows: strain 623, light cycle (*F*-value = 10.89) > light intensity (*F*-value = 0.66) > light combination (*F*-value = 0.11); strain 793, light cycle (*F*-value = 35.67) > light intensity (*F*-value = 4.29) > light combination (*F*-value = 0.13).

After response surface optimization, the theoretical optimal culture scheme for polysaccharide accumulation in *A. platensis* OUC623 and *A. platensis* OUC793 is shown in Table 8. When the light intensity is 31.32 μmol/m^2^s and the light time is 16.00 h, LED light combination is blue: red = 6.24:1, the theoretical maximum value of polysaccharide of strain 623 is 126.60 mg/g. When the light intensity is 31.85 μmol/m^2^s and the light time is 16.00 h, LED light combination is blue: red = 6.08:1, the theoretical maximum value of polysaccharide of strain 793 is 124.73 mg/g. The theoretical values of polysaccharide of strains 623 and 793 are 26.61 and 87.61% higher than those under white LED light, respectively.

#### 3.5.4. The content of protein and phycocyanin

**FIGURE 6:**
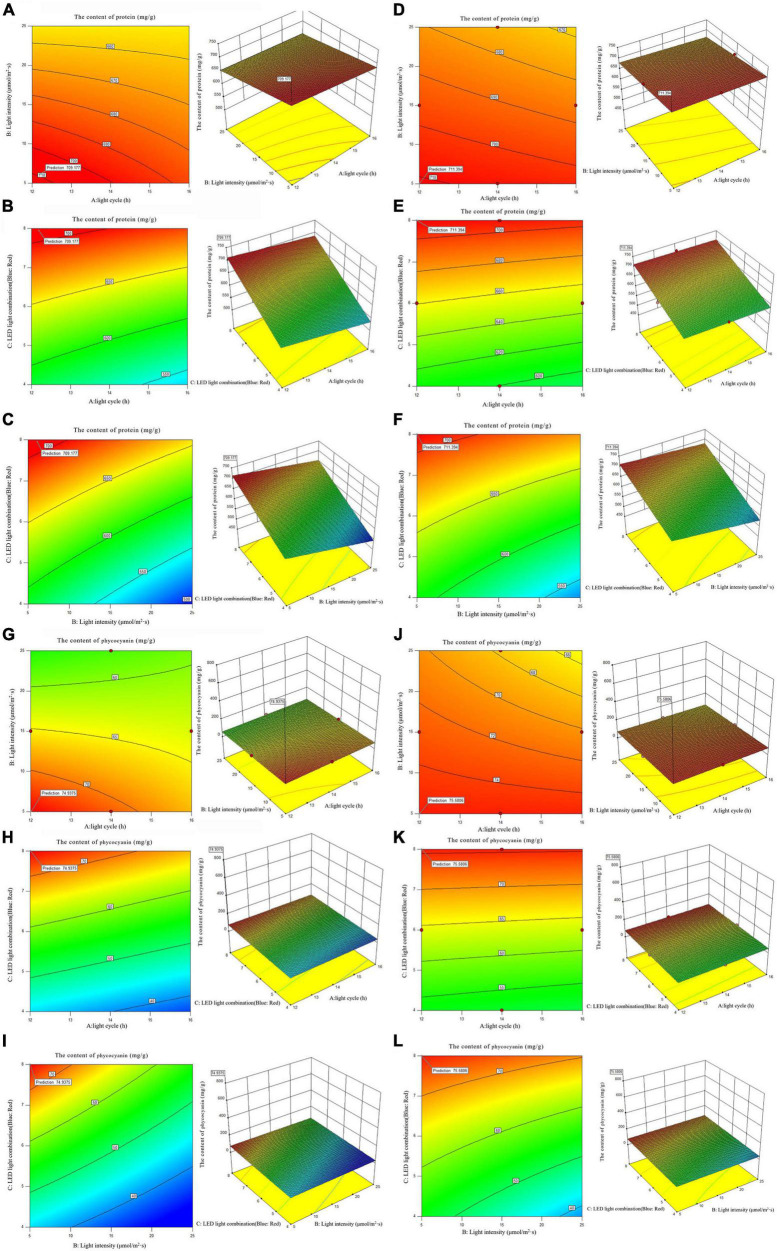
2D contour diagram and 3D surface diagram of protein and phycocyanin content of *Arthrospira platensis* OUC623 and *A. platensis* OUC793. **(A)** Contour diagram and response surface diagram of protein content of 623 with light time (12, 14, and 16 h) and light intensity (5, 15, and 25 μmol/m^2^s) under the light of blue6: red1. **(B)** Contour diagram and response surface diagram of protein content of 623 with light time (12, 14, and 16 h) and red and blue light combination (blue: red = 4:1, 6:1, 8:1) under light intensity of 15 μmol/m^2^s. **(C)** Contour diagram and response surface diagram of protein content of 623 with red and blue light combination (blue: red = 4:1, 6:1, 8:1) and light intensity (5, 15, and 25 μmol/m^2^s) under the light cycle of L14:D10. **(D)** Contour diagram and response surface diagram of protein content of 793 with light time (12, 14, and 16 h) and light intensity (5, 15, and 25 μmol/m^2^s) under the light of blue6: red1. **(E)** Contour diagram and response surface diagram of protein content of 793 with light time (12, 14, and 16 h) and red and blue light combination (blue: red = 4:1, 6:1, 8:1) under light intensity of 15 μmol/m^2^s. **(F)** Contour diagram and response surface diagram of protein content of 793 with red and blue light combination (blue: red = 4:1, 6:1, 8:1) and light intensity (5, 15, and 25 μmol/m^2^s) under the light cycle of L14:D10. **(G)** Contour diagram and response surface diagram of phycocyanin content of 623 with light time (12, 14, and 16 h) and light intensity (5, 15, and 25 μmol/m^2^s) under the light of blue6:red1. **(H)** Contour diagram and response surface diagram of phycocyanin content of 623 with light time (12, 14, and 16 h) and red and blue light combination (blue: red = 4:1, 6:1, 8:1) under light intensity of 15 μmol/m^2^s. **(I)** Contour diagram and response surface diagram of phycocyanin content of 623 with red and blue light combination (blue: red = 4:1, 6:1, 8:1) and light intensity (5, 15, and 25 μmol/m^2^s) under the light cycle of L14:D10. **(J)** Contour diagram and response surface diagram of phycocyanin content of 793 with light time (12, 14, and 16 h) and light intensity (5, 15, and 25 μmol/m^2^s) under the light of blue6:red1. **(K)** Contour diagram and response surface diagram of phycocyanin content of 793 with light time (12, 14, and 16 h) and red and blue light combination (blue: red = 4:1, 6:1, 8:1) under light intensity of 15 μmol/m^2^s. **(L)** Contour diagram and response surface diagram of phycocyanin content of 793 with red and blue light combination (blue: red = 4:1, 6:1, 8:1) and light intensity (5, 15, and 25 μmol/m^2^s) under the light cycle of L14:D10.

According to the analysis of protein and phycocyanin content of single factor experiment, three factors and three levels of light cycle (L12: D12, L14: D10, L16:D8), light intensity 5; 15; 25 μmol/m^2^s), and LED light combination (blue 4: red 1; blue 6: red 1; blue 8: red 1) were selected to culture *A. platensis* OUC623 and *A. platensis* OUC793, respectively. The content of protein was used as the response value. The experimental design and results are shown in Table 7. 2D contour map and 3D response surface map about the effect of experimental factors and their interaction on protein and phycocyanin accumulation of two strains of algae are shown in Figure 6.

##### 3.5.4.1. The content of protein

When the fixed LED light combination is blue: red = 6: 1, the protein accumulation of strains 623 and 793 decreases with the increase light intensity and the light intensity. The interaction between light cycle and light intensity is not significant in strains 623 (*p*-value = 0.5186) and 793 (*p*-value = 0.4607) (Figures 6A, D).

When the light intensity is 15 μmol/m^2^s, the protein accumulation in strains 623 and 793 decreases with the increase of light time and increase with the increase of the proportion of blue light in the LED light combination. The interaction between light cycle and LED light combination is not significant in strains 623 (*p*-value = 0.7046) and 793 (*p*-value = 0.7757) too (Figures 6B, E).

When the light cycle is L14:D10, it is found that the protein accumulation in strains 623 and 793 decreases with the increase of light intensity and increases with the increase of the proportion of blue light in the LED light combination. There is also no significant interaction between light intensity and LED light combination in strains 623 (*p*-value = 0.0790) and 793 (*p*-value = 0.0786) (Figures 6C, F).

The order of primary and secondary effects of light cycle, light intensity and light combination on the accumulation of protein was as follows: strain 623, light cycle (*F*-value = 2.31) > light intensity (*F*-value = 0.78) > light combination (*F*-value = 0.094); strain 793, light cycle (*F*-value = 1.74) > light intensity (*F*-value = 1.58) > light combination (*F*-value = 0.19).

After response surface optimization, the theoretical optimal culture scheme for protein accumulation in *A. platensis* OUC623 and *A. platensis* OUC793 is shown in Table 8. When the light intensity is 6.12 μmol/m^2^s and the light time is 12.18 h, LED light combination is blue: red = 7.95:1, the theoretical maximum value of protein of strain 623 is 709.18 mg/g. When the light intensity is 5.00μmol/m^2^s and the light time is 12.00 h, LED light combination is blue: red = 8.00:1, the theoretical maximum value of protein of strain 793 is 711.39 mg/g. The theoretical values of protein of strains 623 and 793 are 390.88 and 1307.31% higher than those under white LED light, respectively.

##### 3.5.4.2. The content of phycocyanin

When the fixed LED light combination is blue: red = 6:1, the phycocyanin accumulation of strains 623 and 793 decreases with the increase of light time and light intensity (Figures 6G, J). When the light intensity is 15 μmol/m^2^s, it is found that the phycocyanin accumulation in strains 623 and 793 increases with the increase of the proportion of blue light in the LED light combination, decreases with the increase of light time (Figures 6H, K). When the light cycle is L14:D10, it is found that the phycocyanin accumulation in strains 623 and 793 increases with the increase of the proportion of blue light in the LED light combination, decreases with the increase of light intensity (Figures 6I, L).

The interaction among light intensity, light cycle and LED light combination is not significant (*P* > 0.05), which is similar to the results of protein content, The order of primary and secondary effects of light cycle, light intensity and light combination on the accumulation of phycocyanin was as follows: strain 623, light cycle (*F*-value = 1.81) > light intensity (*F*-value = 1.17) > light combination (*F*-value = 0.31); strain 793, light cycle (*F*-value = 0.34) > light intensity (*F*-value = 0.3) > light combination (*F*-value = 0.02).

After response surface optimization, the theoretical optimal culture scheme for phycocyanin accumulation in *A. platensis* OUC623 and *A. platensis* OUC793 is shown in Table 8. When the light intensity is 5.00 μmol/m^2^s and the light time is 12.00 h, LED light combination is blue: red = 8.00:1, the theoretical maximum value of phycocyanin of strain 623 is 75.58mg/g. When the light intensity is 5.01 μmol/m^2^s and the light time is 12.01 h, LED light combination is blue: red = 8.00:1, the theoretical maximum value of protein of strain 793 is 74.94 mg/g. The theoretical values of phycocyanin of strains 623 and 793 are 556.09 and 431.48% higher than those under white LED light, respectively.

### 3.6. Verification experiment of response surface experiment

According to the optimized results of response surface experiment (Table 8), the optimized lighting conditions of strains 623 and 793 were verified, and their growth and organic matter accumulation were measured. The results are shown in Table 9.

**TABLE 9:** Growth rate and organic matter content of *A. platensis* OUC623 and *Arthrospira platensis* OUC793 under optimized light conditions.

	Growth rate	Chlorophyll a content	Carotenoid content	Polysaccharide content	Protein content	Phycocyanin content
*A. platensis* OUC623	0.23 ± 0.02 g/L⋅day	8.91 ± 0.41 mg/g	5.93 ± 0.94 mg/g	312.54 ± 5.12 mg/g	702.32 ± 7.32 mg/g	76.44 ± 0.84 mg/g
*A. platensis* OUC793	0.21 ± 0.01 g/L⋅day	8.03 ± 1.00 mg/g	6.04 ± 0.22 mg/g	274.15 ± 4.12 mg/g	699.97 ± 6.31 mg/g	74.32 ± 2.22 mg/g
623 white LED light	0.12 ± 0.01 g/L⋅day	4.15 ± 1.10 mg/g	3.21 ± 0.34 mg/g	99.99 ± 4.11 mg/g	144.47 ± 2.97 mg/g	11.52 ± 0.44 mg/g
793 white LED light	0.12 ± 0.02 g/L⋅day	3.20 ± 0.87 mg/g	2.83 ± 0.21 mg/g	66.48 ± 2.41 mg/g	50.55 ± 2.21 mg/g	14.10 ± 0.31 mg/g

According to the verification experimental results, the growth rate, chlorophyll a, carotenoid, phycocyanin, protein and polysaccharide contents of strains 623 and 793 compared with white LED light increased by 91.67%, 114.70%, 85.05%, 563.54%, 386.14%, 201.18%, and 75.00%, 150.94%, 113.43%, 427.09%, 1284.71%, 312.38%, respectively.

### 3.7. Expand culture of the two strains in the 20 L photobioreactor using the optimized culture conditions

According to the optimized results of response surface experiment and verification experimental results (Tables 8, 9), the light conditions were set, respectively and the algae strains 623 and 793 were expanded cultured in the 20 L photobioreactor, and their growth and organic matter accumulation were measured.

Verified by cultured in the 20 L photobioreactor, the growth rate, chlorophyll a, carotenoid, phycocyanin, protein and polysaccharide contents of strains 623 were 0.21 ± 0.01 g/L⋅day, 8.55 ± 0.56 mg/g, 5.94 ± 0.45 mg/g, 77.99 ± 2.78 mg/g, 712.42 ± 11.25 mg/g and 301.15 ± 8.44 mg/g, respectively, increased by 77.97%, 106.02%, 85.05%, 476.74%, 384.68%, 201.18% compared with white LED light. For strain 793, the growth rate, chlorophyll a, carotenoid, phycocyanin, protein and polysaccharide contents were 0.22 ± 0.02 g/L⋅day, 8.65 ± 1.21, 6.01 ± 0.14, 70.14 ± 6.88, 684.55 ± 12.44, and 286.11 ± 6.14 mg/g, respectively, which were higher than those of white LED light by 86.96%, 169.47%, 112.37%, 379.36%, 1308.55%, 330.37%, respectively. These results indicated that culture scheme optimized by response surface methodology can be applied to the large-scale culture system of *A. platensis*.

## 4. Discussion

In this experiment, the effect of light cycle, light intensity and LED light combination conditions on *A. platensis* culture at the aspects of the growth rate, chlorophyll a, carotenoid, polysaccharide, phycocyanin and protein content were studied. The results showed that red LED could increase the content of chlorophyll a and carotenoid, and then increase the growth rate of algae. Blue light LED could promote the accumulation of polysaccharide and proteins. In the experiment, the growth rate of strain 623 under the LED light of red: blue = 6:1 was 74.57% higher than that of the white LED under the same condition, the accumulation of chlorophyll a and carotenoid increased by 105.54 and 82.55%, respectively. Under the LED light of blue: red = 6:1, the accumulation of phycocyanin, protein and polysaccharide increased by 346.26, 336.38, and 140.51%, respectively compared with the white LED light. The growth rate of strain 793 under the LED light of red: blue = 6:1 was 62.61% higher than that of the white LED under the same condition, the accumulation of chlorophyll a and carotenoid increased by 157.63 and 98.23%, respectively. Under the LED light of blue: red = 6:1, the accumulation of phycocyanin, protein and polysaccharide increased by 435.18, 1135.71, and 124.97%, respectively compared with those under the white LED light. This is consistent with the trends regarding the effects of red and blue LED light based on photosynthetically active radiation on phycocyanin production of *A. platensis* obtained by Tian et al. (14), Jung et al. (23) and Milia et al. (24). Beside that, Chaiklahan et al. (25) found that in addition to the light intensity, the cell density also affected the content of phycocyanin. It may be due to the increasing self-shading and the decreasing transparency of the algal culture caused by the cell density, which changes the light energy obtained by each cell, so as to change the efficiency of energy consumption. It can be seen that light plays an important role in the growth and organic matter accumulation of *A. platensis*, and it is necessary to optimize the light conditions.

The theoretical growth rate and organic matter accumulation of the two strains were optimized by response surface method. The growth rate, chlorophyll a, carotenoid, phycocyanin, protein and polysaccharide contents of strains 623 and 793 were 91.67%, 114.70%, 85.05%, 563.54%, 386.14%, 201.18%, and 75.00%, 150.94%, 113.43%, 427.09%, 1284.71%, 312.38%, higher than those of white LED light, respectively. All of them were higher than the growth rate and organic matter accumulation of single factor culture. According to the experimental results, the content of chlorophyll a and carotenoid is positively correlated with the growth rate. Under the light conditions with fast growth rate, the content of pigment is higher. In the process of photosynthesis, chlorophyll a and carotenoid play a very important role in light capture, and suitable light conditions will promote the synthesis of chlorophyll a and carotenoid, thus promoting the growth of algae. When the light is insufficient, the contents of chlorophyll a and carotenoid decrease, which is not conducive to light harvesting and photosynthesis; while under the condition of excessive light, *A. platensis* consumes part of its own energy to protect algae cells and photosynthetic system, and even photoinhibition occurs, so that photosynthesis is inhibited. Heber et al. (26) found that under the condition of high light intensity, the central protein (DI) of photosystem II will be consumed, thus reducing the energy transfer efficiency of the light-harvesting complex to photosystem II, so excessive light and lack of light will not be conducive to the growth of algae.

It was found that the contents of polysaccharide and proteins were positively correlated with the proportion of blue LED light. The possible reason is the regulation mechanism of carbonic anhydrase in algae. Blue LED light was proved to promote the transcript of carbonic anhydrase, and then increase the synthesis of carbonic anhydrase (27). The increase of carbonic anhydrase activity is synergistic with the increase of carbonic anhydrase synthesis (28). Carbonic anhydrase is an important enzyme involved in photosynthesis. Carbonic anhydrase can accelerate the diffusion of inorganic carbon to the active site of carboxylase and increase the concentration of inorganic carbon around carboxylase (29). Carbonic anhydrase can increase the carboxylation activity of ribulose 1,5-diphosphate and phosphoenolpyruvate. Ribulose 1,5-diphosphate is an important five-carbon sugar in Calvin cycle. It can immobilize CO_2_ to synthesize an unstable hexaphosphate, which is then decomposed into triphosphoglycerate. Both triphosphoglycerate acid and phosphoenolpyruvate will participate in the process of glycolysis, and the synthesized pyruvate will promote the tricarboxylic acid cycle. The tricarboxylic acid cycle is an important reaction process for the synthesis of polysaccharide, proteins and other organic compounds in organisms, so it will promote the synthesis of polysaccharide, proteins and other organic compounds in algae. Therefore, blue light significantly increases the synthesis of carbonic anhydrase, and then promote the synthesis of organic compounds such as polysaccharide and proteins. According to the conclusion of the stroboscopic experiment of red and blue light on brown algae by Jungandreas et al. (30), when the radiation light is transformed from red light to blue light, it will promote the protein synthesis of algae. This is consistent with our research results in the two strains of *A. platensis.*

Under the condition of short time light and low light intensity, the content of phycocyanin in *A. platensis* increased significantly. As a light-harvesting pigment protein, phycocyanin’s main light absorption region is orange(615–640 nm), which is different from the absorption wavelength of chlorophyll (429–453 nm, 645–663 nm). Therefore, phycocyanin can absorb more wavelength light energy on the basis of chlorophyll-trapping light energy, which makes *A. platensis* better adapt to underwater low light conditions. Markou (13) cultured *A. platensis* with different wavelengths of light (green, blue, red, white, yellow, pink). It was found that the growth rate was the highest under red light and the lowest under blue light. The reason was that the wavelength of red light (622–760 nm) was near the absorption peak of chlorophyll (645–663 nm) and phycocyanin (615–640 nm) at the same time. It was also found that the contents of phycocyanin and chlorophyll were higher at wavelengths that could not provide enough light energy for the growth, such as blue and green. Because the light at these wavelengths could not provide enough light energy, which could be considered as the dark environment for photosynthetic cells, requiring additional synthesis of phycocyanin and chlorophyll to capture light energy. In this experiment, under the condition of insufficient light, the content of chlorophyll a and other pigments directly related to photosynthesis did not increase significantly, but the content of phycocyanin increased significantly, presumably in order to meet the growth of algae under low light. That is consistent with the experimental conclusion that Lee et al. (31) cultured *A. platensis* under LED light to produce high purity phycocyanin. It was speculated that when the light energy absorbed by chlorophyll a and carotenoid cannot meet the growth needs of algae, *A. platensis* is inclined to synthesize more phycocyanin to absorb light in a wider wavelength range to maintain its own growth and organic synthesis.

In addition, the two strains showed different characteristics. The growth rate between the two algae strains was different and strain 623 showed a higher growth rate, which may be related to their different photosynthetic pigment contents. The content of chlorophyll a and phycocyanin in strain 623 was higher than that of strain 793, which made it possible to obtain more light energy for growth. So that the growth rate of strain 623 was higher than that of strain 793. We also found that the two species had different light requirements, strain 623 was more suitable for low light and strain 793 was more demanding for long light time and high light intensity. It may also be related to the high content of chlorophyll a and phycocyanin in strain 623, which may ensure it to capture enough light energy under the low light condition for growth. In addition, perhaps the high pigment content of 623 strain made it more sensitive to light, thus forming a spiral shape to shield each other and reduce the area receiving light. Strain 793 can tolerate high light intensity because of its low content of chlorophyll and phycocyanin, and its linear structure can make it accept more light to meet its growth needs. Gao et al. (32) proved through experiments that high light intensity can cause damage to photosystem II of algae, and *A. platensis* will tighten its helix under high light intensity, which is a self-protective measure for algae to cope with light damage. According to the experiments of Noor et al. (33) and Mühling et al. (34), it was proved that the spiral shape of *A. platensis* changed when the light condition changed, and remained stable for a long time in the process of culture. Similarly, Mao and Guo (35) found that under the combination of red light and red: blue = 8:2, the accumulation of carbohydrate, protein, phycocyanin and oil in *A. platensis* was higher than that in white light, and that the length, diameter and pitch of *A. platensis* were significantly smaller than those in white light. The reason for the analysis is that under the combined red and blue light, the gene expression of *A. platensis* changes, thus tightening the spirals to cover each other to reduce light radiation. Gao et al. (36) found that when the light condition is not suitable for the growth of *A. platensis*, under UV-B, the algae always have the characteristics of shorter length and higher spiral density. In the study, it is found that the algae are exposed to higher UV-B radiation, and their DNA molecules are indeed degraded, which is an important reason for filament fracture. Such morphological changes from loosened to tightened helix appear to be associated with the protective strategy of *A. platensis* to counteract solar UVR.

*Arthrospira platensis* is one of the main algae in microalgae culture. The improvement of the level of *A. platensis* industry is of great significance for the sustainable development of microalgae industry. The strain 623 initially showed the advantage of fast growth, higher chlorophyll a polysaccharide, protein and phycocyanin content in the culture cycle, but its carotenoid content was not as high as that of the strain 793. After optimizing the light conditions, the growth and organic matter content of the two strains were almost the same, and both of them could reach a higher level. So, it is very important to optimize the culture condition for production. Both strains can reach high production level under the optimal culture condition. According to the results of this study, the light conditions can be adjusted according to the actual production needs in the industrial culture of *A. platensis*. LED has the advantages of small size, long life, high efficiency and low energy consumption. In this study, LED light with different red-blue ratio can promote the growth of algae cells and the accumulation of polysaccharide, protein and other organic compounds. The use of LED light can be adjusted according to the demand for products in the production of *A. platensis*, so as to improve productivity, reduce culture energy consumption, achieve the goal of high yield and energy saving, which has economic benefits and ecological value.

## Data availability statement

The original contributions presented in this study are included in the article/supplementary material, further inquiries can be directed to the corresponding author.

## Author contributions

ZX-N designed the research and was involved in interpreting the results and editing the manuscript. SJ-F and SM-H conducted the research. SJ-F and ZX-N analyzed the data and wrote the manuscript and had primary responsibility for the final content. All authors read and approved the final manuscript.
